# Development of a comprehensive measure of reproductive coercion and abuse for global use: a Delphi study

**DOI:** 10.1080/26410397.2026.2652218

**Published:** 2026-04-08

**Authors:** Desireé LaGrappe, Angela Taft, Leesa Hooker, Laura Tarzia, Kristina Edvardsson

**Affiliations:** a Lecturer & PhD Candidate, Judith Lumley Centre, School of Nursing and Midwifery, La Trobe University, Bundoora, Victoria, Australia; PhD Student, SPHERE Centre of Research Excellence in Women’s Sexual and Reproductive Health in Primary Care, Department of General Practice, School of Public Health and Preventive Medicine, Monash University, Melbourne, Australia.; b Emeritus Professor, Judith Lumley Centre, School of Nursing and Midwifery, La Trobe University, Bundoora, Victoria, Australia; Associate Investigator, SPHERE Centre of Research Excellence in Women’s Sexual and Reproductive Health in Primary Care, Department of General Practice, School of Public Health and Preventive Medicine, Monash University, Melbourne, Australia; c Professorial Research Fellow, Violet Vines Marshman Centre for Rural Health Research, La Trobe Rural Health School, La Trobe University, Bendigo, Victoria, Australia; Professor, Judith Lumley Centre, School of Nursing and Midwifery, La Trobe University, Bundoora, Victoria, Australia; Research Affiliate, SPHERE Centre of Research Excellence in Women’s Sexual and Reproductive Health in Primary Care, Department of General Practice, School of Public Health and Preventive Medicine, Monash University, Melbourne, Australia; d Professorial Fellow, Department of General Practice & Primary Care, The University of Melbourne, Carlton, Victoria, Australia; Professor, Centre for Family Violence Prevention, The Royal Women’s Hospital, Parkville, Victoria, Australia; Chief Investigator, SPHERE Centre of Research Excellence in Women’s Sexual and Reproductive Health in Primary Care, Department of General Practice, School of Public Health and Preventive Medicine, Monash University, Melbourne, Australia; e Professor, Judith Lumley Centre, School of Nursing and Midwifery, La Trobe University, Bundoora, Victoria, Australia; Chief Investigator, SPHERE Centre of Research Excellence in Women’s Sexual and Reproductive Health in Primary Care, Department of General Practice, School of Public Health and Preventive Medicine, Monash University, Melbourne, Australia

**Keywords:** reproductive coercion, intimate partner violence, sexual violence, induced abortion, patient reported outcome measure, Delphi method

## Abstract

Reproductive coercion and abuse describes controlling behaviours to undermine another person's reproductive autonomy, predominantly affecting women and gender minorities with pregnancy capacity. Debate exists about how this form of gender-based violence should be measured and whether available measures developed over a decade ago adequately capture current conceptual understandings. To address this, our study aimed to develop a comprehensive measure of reproductive coercion and abuse for global population-level use. A modified e-Delphi technique was used to determine consensus on measurement items generated from pre-existing literature and formative qualitative research. Global experts (*n* = 30) with research, clinical, and/or lived expertise were recruited from low- to middle- and high-income countries and asked to rate items against COSMIN criteria. Participants represented 15 countries and five global regions (Africa, Asia, Europe, North America, Oceania). They suggested 12 new items in addition to the 62 candidate items. Consensus was not reached on 17 of 74 items. After incorporating final feedback, 59 items were selected for future pilot testing. The Delphi panel did not reach consensus on structure of response options or how to ask about intent. Experts agreed measuring the distinct interpersonal relationships in which reproductive coercion and abuse can occur is important. We developed a robust set of new items to measure reproductive coercion and abuse within multi-country comparative studies that fills a measurement gap in sexual and reproductive health. With future psychometric evaluation, the measure will enable more sensitive, specific, and consistent prevalence estimates across contexts, critical to inform practice and policy locally and globally.

## Plain Language Summary

The use of pressure, threats, or force to control someone else's choices about birth control, pregnancy, or abortion is called reproductive coercion and abuse. It mainly affects people who can become pregnant. There is debate about how to ask about this problem in surveys. Current surveys were created more than ten years ago, when our understanding was new. We now know more about the different behaviours that can be used. To help improve measurement by including all types of this abuse, we created a new survey. We asked researchers and clinicians around the world for their opinions about the survey using the Delphi method, a research process to find out if experts agree. The survey included questions from previous research and new questions suggested by people with first-hand experience of reproductive coercion and abuse. Thirty experts from 15 countries across Africa, Asia, Europe, North America, and Oceania took part in our study. The experts added 12 new questions to our survey for a total of 74 questions but disagreed on 17. Experts agreed asking about the type of relationship where the abuse occurs is important. They disagreed on response choices and how to ask about the motivation behind controlling behaviours. At the end, 59 questions were chosen for next steps. Our survey fills a gap in sexual and reproductive health research. With more testing, the survey will improve the accuracy of measuring this complex problem around the world. This information is critical to inform local and global practice and policy solutions.

## Resumen

La coerción y el maltrato reproductivos son el control de comportamientos para socavar la autonomía reproductiva de otra persona, que afecta predominantemente a mujeres y minorías de género con capacidad gestante. Se debate cómo se debe medir esta forma de violencia de género y si las medidas disponibles creadas hace más de una década capturan adecuadamente la comprensión conceptual actual. Para abordar estas interrogantes, nuestro estudio buscó crear una medida integral de la coerción y el maltrato reproductivos para uso a nivel de población mundial. Se utilizó la técnica e-Delphi modificada para determinar consenso sobre los ítems de medición generados de literatura preexistente e investigación cualitativa formativa. Se reclutaron expertos mundiales (*n* = 30) con experticia en investigación, clínica y/o vivida provenientes de países de bajos a medianos ingresos y de países de altos ingresos, y se les pidió que calificaran los ítems según los criterios COSMIN. Las personas participantes representaban 15 países y cinco regiones mundiales (África, Asia, Europa, Norteamérica, Oceanía). Sugirieron 12 ítems nuevos además de los 62 ítems candidatos. No se llegó a un consenso en 17 de los 74 ítems. Después de incorporar los comentarios finales, se seleccionaron 59 ítems para futuras pruebas piloto. El panel Delphi no llegó a un consenso sobre la estructura de las opciones de respuesta ni sobre cómo preguntar acerca de la intención. Los expertos estuvieron de acuerdo en que es importante medir las diferentes relaciones interpersonales en las que puede ocurrir coerción y maltrato reproductivos. Creamos una serie robusta de ítems nuevos para medir la coerción y el maltrato reproductivos en estudios comparativos multinacionales, que llena la brecha de medición en salud sexual y reproductiva. Con una futura evaluación psicométrica, la medida permitirá realizar estimaciones de prevalencia más sensibles, específicas y consistentes en diferentes contextos, lo que es fundamental para informar la práctica y las políticas a nivel local y mundial.

## Résumé

Le terme de coercition et maltraitance reproductives désigne des comportements de contrôle visant à compromettre l’autonomie reproductive d’une personne, qui affectent principalement les femmes et les personnes de minorités de genre pouvant mener à bien une grossesse. des débats portent sur la manière de mesurer cette violence sexiste et la pertinence des mesures actuelles, élaborées il y a plus de dix ans, pour tenir compte fidèlement des conceptions actuelles. à cette fin, notre étude visait à mettre au point un outil de mesure complet de la coercition et de la maltraitance reproductives, applicable à l’échelon mondial. une méthode delphi modifiée en ligne a été utilisée pour dégager un consensus sur les éléments de mesure, issus de recherches qualitatives formatives et de publications préexistantes. des experts mondiaux (n = 30) possédant une expertise clinique, vécue et/ou en recherche ont été recrutés dans des pays à revenu faible, intermédiaire et élevé, et ont été invités à noter les éléments par rapport aux critères cosmin. les participants représentaient 15 pays et cinq régions du monde (afrique, asie, europe, amérique du nord et océanie). ils ont suggéré 12 nouveaux éléments en plus des 62 éléments candidats. aucun consensus n’a été atteint sur 17 des 74 éléments. après inclusion des derniers retours, 59 éléments ont été sélectionnés pour être testés ultérieurement dans une phase pilote. le groupe delphi n’est pas parvenu à un consensus sur la structure des options de réponse ni sur la manière d’interroger les personnes sur leurs intentions. les experts ont cependant convenu qu’il est important de mesurer les différentes relations interpersonnelles au sein desquelles la coercition et la maltraitance reproductives peuvent se produire. nous avons élaboré un ensemble solide de nouveaux éléments pour mesurer la coercition et la maltraitance reproductives dans le cadre d’études comparatives multinationales, comblant ainsi une lacune dans les mesures de santé sexuelle et reproductive. avec une évaluation psychométrique ultérieure, l’outil de mesure permettra des estimations de prévalence plus sensibles, spécifiques et cohérentes selon les contextes, un point essentiel pour guider les pratiques et les politiques aux niveaux local et mondial.

## Introduction

Reproductive coercion and abuse (RCA) describes controlling behaviours that undermine autonomy in reproductive health decision making, including use of relentless pressure, threats, or physical and sexual violence to force another person to have an unwanted pregnancy or abortion.^1^ This distinct form of gender-based violence (GBV) was first described as “reproductive coercion” in 2010 by Miller and colleagues and predominantly affects women and gender minorities with the capacity to become pregnant.^2^ RCA overlaps with domestic, family, and sexual violence (DFSV), but lacks a consistent definition and measurement framework.^1^ This form of abuse, entangled with gender and societal expectations regarding childbearing, greatly impedes the full realisation of sexual and reproductive health and rights (SRHR) globally. The antithesis of reproductive autonomy, RCA violates SRHR by interfering with individuals’ (often women and girls) as well as couples’ ability to freely choose – without coercion or violence – whether or not to parent and the number, spacing, and timing of children.^3^ RCA impedes gender equality and disability rights through coerced and forced selective reproduction and within the context of marital rape and early marriage.^4–6^ RCA can be perpetrated by male partners or guardians, as examples, as well as other gatekeepers, by hindering access to SRH information and services, or in cases of incest, by forced abortion by the perpetrator or other relatives to hide the abuse.^7^ RCA has been linked to deprivation of liberty and can have long lasting impacts on educational and economic opportunities, as well as sexual wellbeing and future relationships.^8–10^ More broadly, RCA falls under the umbrella “reproductive violence”, which constitutes gender persecution under international law and includes obstetric and gynaecological violence (i.e. health worker and institution/ systems level violations over SRH autonomy), denial of abortion care, and conflict-related violence, such as genocidal rape.^7^

Although RCA prevalence is likely imprecise due to current measurement inconsistencies and limitations, estimates can range from 2% to over 20% depending on the cohort, context, and subdomain(s) measured (e.g. pregnancy coercion vs a full spectrum of behaviours).^11,12^ Studies of varying quality suggest RCA is directly associated with higher rates of unintended pregnancies and adverse mental, sexual and reproductive health (e.g. post-traumatic stress disorder; sexually transmitted infections), and possibly broader maternal and child health outcomes, including parenting.^6,8,10,11^ RCA often occurs within intimate partner and close family relationships (e.g. parents; in-laws), as well as broader community social networks, particularly where reverence for religious leaders and elders and obligations to community are paramount.^6,13^ RCA behaviours can manifest differently across contexts and cultures, creating challenges when attempting to comprehensively and reliably measure the concept with limited item numbers.^12,13^

Despite fluctuations in geopolitical relations and commitments to health and gender equity, the aim to meet Sustainable Development Goal (SDG) targets to reduce GBV alongside reducing unplanned and unwanted pregnancies has been a shared priority to improve health for all.^14^ RCA has gained increasing global attention by clinicians, researchers, and policy makers alongside increasing awareness that those who experience DFSV are less likely to have control over when, or if, they have sex or use contraception.^14,15^ While public health policies and interventions to address RCA advance to scale, leading experts in the field argue that neither an internationally accepted and consistent definition, nor a comprehensive, validated measure are available.^1,16^ As with other areas of public health, lack of standardisation threatens our ability at local and global levels to fully understand and therefore appropriately address RCA (e.g. measure the effectiveness of interventions). This is especially the case for RCA given its complex intersection with other forms of GBV, in addition to ubiquitous, repro-normative pressures and pro-natalist policies.^1^

Most of our early understandings about RCA originated from family planning settings in the United States of America (US) and current knowledge remains largely influenced by how RCA has been measured using US-based scales. These measures are predominantly Miller's Reproductive Coercion Scale, the most widely cited and specific, measuring pregnancy coercion and contraceptive sabotage^2,16^ and the Reproductive Autonomy Scale, the second most widely cited, measuring freedom from coercion alongside broader domains (e.g. communication).^17^ While groundbreaking in informing early understandings of RCA, these older measures noticeably omit items about abortion coercion and abuse, have been critiqued for conflating sexual violence and RCA, issues likely affecting their sensitivity and specificity, and have had varied success in their global application.^1,12,18,19^ Their varied global applicability may reflect that they are unable to capture recognised differences in social norms that influence reproductive decision-making across countries. The order of how these scales were validated in the USA first and then adapted internationally, however, is not uncommon. In international development generally, measurement tools and interventions are routinely developed and validated first in high-income countries. They are then retrofitted through adaptation to other cultural contexts, versus research innovation exchange in the other direction.^20^ As we shift towards more equitable and decolonising approaches to global health, new measurement tools have been developed that are increasingly internationally collaborative and inclusive in earlier stages of development, rather than post hoc, including SRHR tools intended for multi-country comparative studies.^21,22^

In addition to being constrained by conceptualisations of reproductive decision-making and abuse originating among US cohorts, these widely applied and validated scales do not reflect newer conceptual and cross-cultural insights because of the timing of their development. RCA conceptual understandings have only begun to reach sufficient publication numbers to warrant the first qualitative evidence synthesis published in 2021 ^23^ and as of 2025, the first dedicated review of first-hand survivor experiences that identified 58 papers.^10^ This is a near five-fold increase in the number of qualitative or mixed methods RCA studies identified by Grace et al.'s 2018 landmark review.^11^ There has also been a noticeable surge in the number of both conceptual, qualitative and quantitative studies published outside of the USA since 2020 (i.e. COVID-19 pandemic).^6,16,24–26^ By comparison, the pioneering Reproductive Coercion Scale was developed in 2010,^2^ and refined and validated in 2017.^16^ The Reproductive Autonomy Scale was developed and validated in 2014.^17^ RCA measurement has fallen behind in reflecting newer conceptual and cross-cultural knowledge. Integration of more recent research into current RCA measurement would serve to enhance how we address this significant public health problem both within and across diverse country contexts.

### Aim

We sought to develop and assess the content validity of a new globally relevant, rigorous and comprehensive RCA outcome measure for multi-country prevalence studies and comparison across cultural contexts. By including all RCA domains and greater nuance, the measure aims to more sensitively and specifically estimate RCA prevalence among women and gender minorities with current or past capacity to become pregnant.

## Methods

**Figure 1.**
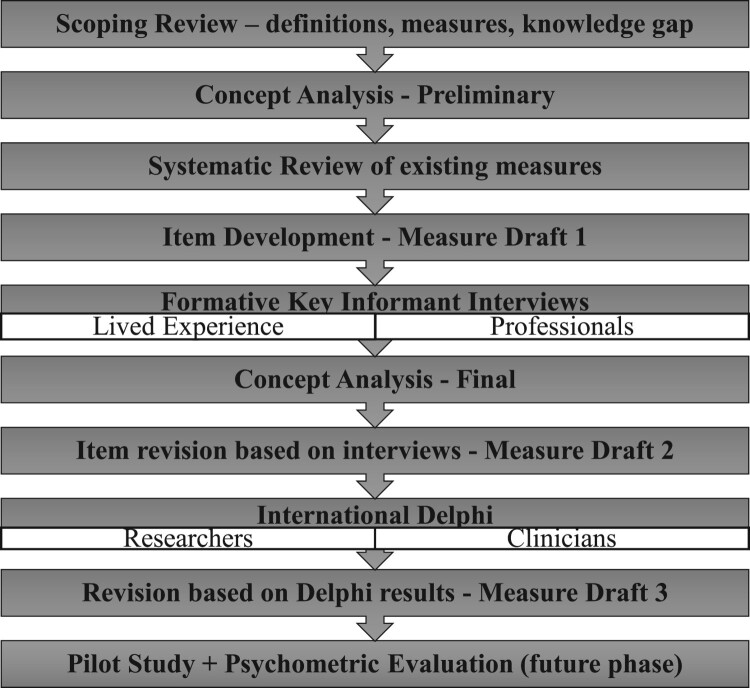
Development process

This research study was part of a larger multi-phase project using exploratory sequential mixed methods to develop and assess the content validity of a new comprehensive RCA measure, building on prior global research. The process of development, outlined in Figure 1 and explained in more detail below, involved (i) an extensive review of the literature; (ii) a qualitative phase; (iii) the present Delphi study (focus of this paper); and (iv) will include pilot testing (e.g. cognitive interviewing) and psychometric evaluation (future research). We report our research in accordance with DELPHI studies in social and health sciences – recommendations for STAndardized Reporting (DELPHISTAR).^27^

### Research in previous phases

The present study is built on a systematic review of the literature^28^ and consultation with lived experience experts and health, social service, and advocacy professionals born in ten different countries and residing in Australia (submitted in full elsewhere).^29^ The aims of the literature review were to (i) identify the breadth and scope of RCA behaviours and their measurement globally and (ii) determine whether development of a new measure was justified by evaluating the measurement properties of validated RCA measures. The subsequent formative qualitative study sought to refine the conceptual understandings and boundaries of RCA for measurement purposes and seek feedback on draft items derived from the literature review.^29^

Through the previous research phases, we identified several key areas for improvement within current RCA measures presented in Table 1. The Delphi study that this paper reports on focused on refining the draft measure developed during the qualitative phase, by amalgamating global perspectives and expertise.

**Table 1.** Key areas for improvement or clarification in RCA measurement identified through previous research phases

Findings and sources	Implications for improvement or clarification
Literature review **Control and abuse over abortion decision-making is a core RCA domain** in both intimate partner and dating violence yet missing from most validated scales.^24,25,30^ Qualitative analysis Nearly half of the lived experience participants disclosed abortion coercion and abuse (to force and/or obstruct) and providers spoke frequently about abortion's role. **Abortion stigma was identified as an influencing factor**.	**Add control over pregnancy outcomes**, including abortion coercion and abuse, as a domain.
Literature review **Categories of abusive actors** (i.e. perpetrators) **and relationship types measured** (e.g. intimate partners, relatives) are **inconsistent across geographical locations**, despite a globalised world with increasingly diverse, multicultural/ diaspora societies.^28^ Qualitative analysis RCA was perpetrated by both intimate partners and family members for half of the lived experience participants across demographic groups.	**Consistently measure whether RCA is perpetrated by intimate partners, family relatives, or both in all contexts** (high-, middle- and low-income countries).
Literature review Voices of **key populations advocating for action on RCA are often missing** from the design of widely used measurement scales (e.g. migrant/refugee and Indigenous women; people living with a disability and/or HIV; LGBTQIA+ communities).^31,32^ A critique of some IPV measures is that situational, social, cultural, and historical contexts matter for measurement, but can be missing.^31,33^ Qualitative analysis **Cultural contexts** (e.g. religious beliefs, norms surrounding sex or contraception) **and structural contexts** (e.g. political and health systems that can limit reproductive choices) were **identifed as key themes “interlinked” with RCA**.	**Include and amplify missing marginalised voices** in RCA measurement development. **Item wording should consider cultural and structural contexts** and aim to tease out abusive behaviours enabled by these norms, beliefs and systems’ abuses.
Literature review There is **conflicting literature about RCA's conceptual boundaries**. At one end, RCA is described as synonymous with obstetric and gynecological medical coercion or state sponsored reproductive violence.^34,35^ At the other end, some scholars argue interpersonal RCA is distinct from institutional and structural forms.^1^ Despite this debate in the academic sphere, no research had deconstructed the conflicting viewpoints with lived experience experts. Recent measurement tools on coercive practices in SRH care provision suggest that medical coercion, while closely related, is its own construct.^36,37^ At the time of our research, it remained unclear whether there was global expert consensus on whether one scale should measure the spectrum of reproductive autonomy violations across a socio-ecological model.	**Deconstruct with lived experience experts the conflicting conceptual viewpoints** of whether interpersonal RCA is a distinct phenomenon versus a synonymous concept with SRH related medical coercion. **Aim to establish global expert consensus** on whether one scale should measure the spectrum of reproductive autonomy violations across a socio-ecological model.
Literature Review Within broader GBV prevention, holding those who use violence accountable is critical to shift the focus from ‘victim blaming’.^38^ Within measurement tools, **asking about the abusive actor and their behaviourally specific acts is the recommended standard**.^39^ Qualitative analysis Findings suggest **solely asking about the abusive actor and their behaviourally specific acts, not the viewpoint of the affected person, may have unintended consequences**. Participants stated that repetitive item wording asking “Has [perpetrator x] ever done [y behaviour] to you?” could inadvertently reinforce male-dominance and their limited control within their relationship, or their feelings of limited self-worth. This was described as having the potential to reduce accurate reporting and was specifically raised by those entwined in coercively controlling or emotionally manipulative relationships that foment self-doubt, or who feared retribution from partner(s), family and/or authorities. They suggested instead to invert the questions to ask, “Have you ever been [behaviour y] by [perpetrator x]?” which could invite deeper reflection on their personal experiences and accounts of events versus the abusive person's narrative.	**Consider adopting item wording with structure “Have you ever been [behaviour y] by [perpetrator x]?” and test in parallel for comparison with standard item wording**, e.g. “Has [perpetrator x] ever done [y behaviour] to you?”
Literature review Response options for the most cited measure, the Reproductive Coercion Scale, among others, are binary (Yes/No).^16^ **In scale development theory, most attitudes and behaviours lie on a continuum**, resulting in lost information when participants, who do not see the response as binary, are ‘forced’ to respond either/or.^40^ In reality, while a behaviour has happened or not, someone within an abusive relationship experiencing surreality due to unrelenting emotional manipulation, or with brain injury symptomatology from physical violence, may not have clarity around the certainty of that event.^41,42^ Those with lived experience, particularly those currently experiencing abuse, may judge their situation based on the severity of previous instances of abuse, or by comparison to others’ experiences or media representations (e.g. obvious physical injury; femicide). Undermining by the abusive person can also distort their reality, blurring lines with the abusive person's false narratives.^41^. Allowing for **continuous responses creates room to indicate an oscillating or hesitant ‘yes’** where uncertainty exists, rather than forcing an inaccurate ‘no’ response.^40^ Qualitative analysis “In my head”: emotional manipulation and deceit could make answering questions about intent of RCA behaviours or certainty of events difficult.	**Use continuous response options and pilot test** to allow for oscillating or hesitant ‘yes’ responses. **Consider separating out perpetrator intent from the parent item** and making adjustments in scale scoring.

### Study design

A modified Delphi technique conducted electronically (e-Delphi) between 29 July – 09 October 2024 was used to develop the measure using global expertise to find consensus on items for inclusion.^43^ The study design followed an a priori protocol, as described below and submitted to ethics prior to the conduct of the research. The Delphi technique is commonly used in health care research to generate consensus on difficult to define, under-researched concepts that are context and expertise specific, and ethically or logistically difficult to study, therefore highly suited to RCA.^44^

#### Predetermining core concepts and questionnaire items

We conducted a systematic search of the literature to identify core RCA concepts, previously validated questionnaires, and other survey items consistent with RCA, as well as key knowledge gaps. We drafted an initial item list using the findings and presented them for feedback during the qualitative phase to lived experience and professional experts (*n* = 30), as described above. Through interviews and focus groups, guided by COSMIN criteria, we (i) explored congruence between participants’ experiences and the initial item list to enhance relevance and comprehensiveness, and (ii) asked for opinions about how best to elicit accurate responses and enhance comprehensibility.^29^ Results were analysed prior to Round 1 of the present Delphi study and used to refine the proposed 62 candidate items presented to the expert panel. We chose to use predetermined items rather than open-ended questions, characteristic of traditional Delphi studies, to (i) prioritise lived experience and professional expertise collected during the qualitative phase, (ii) acknowledge the existing foundation of prior research by experts whom we aimed to recruit, and (iii) minimise participant fatigue.^43^

#### Delphi panel

##### Selection of expert panel

As there is no consensus on the definition of “expert”,^43^ we defined “expert” as someone who had:

- advanced clinical or professional experience in advocating for and/or responding to clients or patients experiencing RCA and/or
- authored or contributed to published, peer reviewed research, continuous quality improvement projects, organisational policies/programmes, grey literature and/or white papers about RCA and/or
- lived experience of RCA;
  AND (clinicians and researchers only)
- acknowledged current survey methodological issues; and was
- recognised among their peers as knowledgeable on the topic;

Desirable criteria included:

- experience in survey development and design;
- being recognised as a global leader in the field of GBV, and more specifically RCA, as evidenced by invitations to give keynote presentations or consult for peak bodies, for example.

To increase validity, we sought to select an international heterogeneous panel including experts in GBV and sexual and reproductive health.^45^ We aimed to recruit participants born in and working across global regions and country income group classifications (low-, middle- and high-income), as well as professional backgrounds, including those with lived experience. We considered how we could increase inclusion of those who identify with and/or advocate on behalf of underrepresented, or marginalised, demographic groups (e.g. Aboriginal and Torres Strait Islander, Living with Disability, migrant/refugee, LGBTQIA+, adolescents and youth, etc.). Our evidence review identified that individuals from these groups are less likely to be included in research but may have heightened risk of RCA.^5,31,32,46^

We purposively recruited experts from researchers identified through the peer reviewed and grey literature on RCA, and the professional networks of the primary investigatory team, in addition to referred experts (i.e. snowballing). Experts were approached by email, telephone, and face-to-face. To handle missing knowledge, expertise, and perspectives, we reviewed participation after each round to ensure the panel's composition remained as originally intended.^43^

Selected panellists remained anonymous to each other during and after the study. Their details were not openly communicated and their responses were anonymised (i) given the relatively small, niche research field; and (ii) to reduce bias or influence caused by power differentials like perceived expert seniority or other group dynamics (e.g. groupthink, social desirability bias).^44^

At the time of recruitment, participants were offered up to $100AUD for their contributions or an equal donation made anonymously on their behalf to an Aboriginal Community Controlled Organisation providing DFSV services.

#### Determination of panel size

We planned to approach 30 experts with the aim to recruit 12–15 experts in order to account for possible attrition (20–30% dropout anticipated between rounds) and establish a quorum for each round of 7 (roughly 50%), considered the minimum panel size.^43^

#### Questions for Delphi panel

Questions to solicit expert opinion on candidate items developed from the formative phase were informed by COSMIN Guidelines.^47^ To reduce research burden on panellists, COSMIN criteria were condensed using single items to rate the relevance (i.e. “How would you rate the relevance of this item?”) and comprehensibility (i.e. “How would you rate the comprehensibility of this item?”). Relevance covered the construct (RCA), population (individuals considered at current or past “risk” of pregnancy regardless of gender identity or sexuality, including those who experience infertility), and context (population measure applicable in any general community or health care setting). Comprehensibility covered item wording and appropriateness. For every main item of the measure, participants were first asked to give a summative response considering the criteria for relevance and second, considering the criteria for comprehensibility.

As a single item pertaining to all items collectively, panel participants were asked separately to give a summative score about the response options considering relevance (appropriateness, understanding as intended, recall period) and comprehensibility (match with item). They were also asked about the comprehensibility of the measure's preamble and overall comprehensiveness of the measure (e.g. Are all key concepts included?).

Panellists were asked to use a 10 -point sliding scale to rate criteria, with an option to score zero for not relevant and a mid-point of five for neutral responses (i.e. a 9-point Likert scale with null value). An optional free-text box was included to provide rationale for their selection, as well as suggestions for missing items, and wording changes. All survey rounds were administered using REDCap^48^ within a two-week timeframe with reminders sent and extensions provided on request to manage non-response.^49^

#### Controlled feedback

Controlled feedback, a classic Delphi characteristic, describes data analysed from each survey round that is selected by the moderator and presented in a simplified format for panel experts to consider.^44^ Prior to each iterative round, panellists received controlled feedback in a de-identified format of medians and interquartile ranges (IQR), as well as open-ended response summaries, aggregated across all expert groups. Experts also received their individual responses relative to the group. Feedback included dissent and mixed results for evaluation in subsequent Delphi rounds. Participants received controlled feedback by embedding their previous answers into the next survey round. For example, next to the survey prompts for each item, participants received their previous scores alongside the de-identified, summarised statistics and open-ended responses. To aid in achieving consensus, controlled feedback served to promote critical reflection on previous responses and careful consideration of subsequent responses.^43^

#### Delphi panel rounds

Prior to Round 1, Delphi participants were briefed with the study aim, protocol procedures, and a summary of our findings from previous research phases. Participants were asked to watch a brief video created by the authors summarising this information for the purposes of:

- limiting confusion by increasing transparency and
- providing a foregrounding in the conceptual basis underpinning this work.

Each subsequent Delphi round was conducted the same as Round 1. Item wording for subsequent survey rounds was amended per feedback from the previous round. All panellists were asked to consider the controlled feedback prior to answering the next round.

#### Termination of Delphi panel

A maximum of three rounds was set *a priori* to reduce survey fatigue and attrition. While traditionally Delphi rounds are iterated until consensus is achieved, changes past three rounds are unlikely.^43,50^

### Statistical analysis

Panel round data, including partial responses, were weighted equally and reported as medians and IQRs.^51^ Statistical analysis was conducted using R Studio.^52^ For transparency we report:

- Percentage response rates including those voting 4 or below and 6 and above;
- Percentage agreement for each item (number voting 7 or above/total participants); and
- Median and range.

#### Consensus and stability

Consensus and stability are considered the most robust analysis of Delphi panel data.^44^ Consensus for each item was calculated after each round using IQRs.^51^ With a 9-point Likert scale, our predetermined criteria for consensus was set at an IQR of two or less.^53^ Stability was calculated after the second and third rounds using the non-parametric Wilcoxon matched-pairs signed rank test, well suited for comparison of Likert scores and handling partial response data.^51^ Stability was achieved when no statistically significant change (*p* <0.05) was detected between rounds.^51^

### Analysis of open-ended responses

Free-text responses were analysed using qualitative content analysis with similar ideas grouped together, condensed, and reported in summary, in addition to providing controlled feedback on unique participant responses.

### Development of final questionnaire

After the final Delphi round, all items that reached consensus and stability with a median Likert score above six were included in the final questionnaire. An exception was made to include items even when:

- there was consensus to remove an item or
- consensus could not be reached

for any item(s) that was/were clearly endorsed by a majority of participants during the qualitative phase and/or by a majority of robust studies identified during the literature review. These items will await psychometric evaluation with larger sample sizes to account for the relatively small number of Delphi participants and recognition that clinically meaningful items do not always meet statistical criteria. This was determined by the primary investigatory team closest to the data and reported in the final results.

### Method consulting

We were advised by experts in the Delphi technique (University of South Australia; University of Melbourne; La Trobe University), latent variable measurement (Johns Hopkins University), and statistics (La Trobe University Statistics Consultancy Platform) in the development of our protocol and data analysis.

### Reflexivity

Researchers of the study group were born in the USA, Australia, and Sweden and are resident in Australia. The research, however, was primarily conducted whilst the primary author, responsible for data collection and analysis, resided in Vietnam, expanding the study's recruitment network in the region. We are all applied researchers who have backgrounds in nursing, midwifery, sociology and public health with extensive mixed-methods research experience. Our work is predominantly in the areas of SRHR and GBV, with a focus on primary prevention, early intervention, and health systems responses. All researchers identify as cisgender White and/or Hispanic women. The primary author has fifteen years research experience, and all other authors are mid-career and senior research academics. We applied an intersectional feminist lens and reproductive justice framework throughout all research phases recognising that our understandings of RCA may be limited by our “Western” worldviews and relatively privileged positionalities. For this reason, rather than progress to develop and test our measure following our qualitative phase, we conducted this Delphi for further triangulation with global perspectives to strengthen the measure's content validity.

### Ethics approval

Ethical approval for this research was granted from the La Trobe University Human Ethics Committee [HEC24252; Approval Period: 24 July 2024 – 2029]. Informed written consent provided electronically was received from all participants. Acknowledging the common lived experience of abuse, particularly among those who choose to work or conduct research on the topic, we embedded trauma- and violence-informed practices throughout.^54^

## Results

### Participants

Of the experts approached (*n* = 39), 30 consented to participate (77% response rate). Experts were from 15 different countries and currently residing in 5 geographical regions (Australia, Europe, North America, Southeast Asia, and Sub-Saharan Africa). Of those who reported their demographic data (*n* = 27), a majority were born between 1981 and 1996 (44%) and were cisgender women (89%) with more than half (55%) describing themselves as from a historically marginalised background. Eight (30%) reported lived experience of RCA and/or sexual violence. The mean years of professional experience within the GBV, RCA and/or SRHR field was 12.7 years with most experts either researchers and/or health care workers. Further demographic details can be found in Table 2.

**Table 2.** Delphi panel demographics

**Demographics**	*n* = 27^1^ (total sample *n* = 30)					
**Year of Birth**	**Age Range**	***n***	**Sex**	***n***	**Gender**	***n***
** **	1946–1964	3	Female	24	woman	24
** **	1965–1980	10	Male	2	man	2
** **	1981–1996	12	prefer not to answer	1	non-binary	1
** **	1997–2006	2				
**Lived experience**	**GBV**		***n***	**Diversity/ Historical marginalisation**		***n***
** **	RCA					
** **	Yes		**7**	Yes		15
1** **	No		18	No		11
** **	Prefer not to answer		2	Prefer not to answer		1
** **	Sexual Violence**		1	Self-reported identity descriptor*		** **
** **				Migrant/refugee background		6
** **				LGBTQIA+		6
** **				Racial minority		3
** **				Aboriginal/Indigenous		2
** **				Living with disability		2
** **				Other/NR		2
**Region**	**Region of residence**		***n***	**Region of birth**		***n***
** **	**High-income**		**23**	**High-income**		**17**
** **	Australia		11	Australia		7
** **	N. America		6	N. America		7
** **	Europe		4	Europe		2
** **	** **		** **	E. Asia		1
** **	**Low to middle-income**		**5**	**Low to middle-income**		**10**
** **	Sub-Saharan Africa		2	Arab Peninsula/ N. Africa		2
	S.E. Asia		3	Sub-Saharan Africa		2
				S.E. Asia		3
** **	**Overlap**		**1**	E. Asia		1
				S. Asia		1
				Latin America		1
**Expertise**	**Years of experience**		x̄, *s* (range)	**Global health experience**		***n***
** **			12.7 (1.7, 1.5–30)			13
	Profession*			**Countries of work experience:**		
	**Researcher**		**23**	Australia, Bangladesh, Botswana, Canada, Denmark, Ethiopia, Eswatini, Egypt, Fiji, India, Ireland, Japan, Kiribati, Kenya, Mongolia, Myanmar, Nepal, Netherlands, Philippines, Timor-Leste, Tanzania, South Africa, Marshall Islands, Morocco, Papua New Guinea, Samoa, Solomon Islands, Sri Lanka, Thailand, Tuvalu, Uganda, UK, USA, Vietnam		
	**Health care worker**		**11**			
	Nurse and/or Midwife		5			
	Medical Doctor		3			
	Social Worker/ Psychologist		2			
	Health Administrator		1			
	**Other**		**8**			
	Advocacy		3			
	Program and Policy		2	** **		
	Teaching academic (nursing/public health)		2	** **		
	Women's legal services		1	** **		

^1^ *n* = 3 demographic data missing – either elected not to report (responses were optional) or did not reach the end of the first survey containing demographic questions; *not mutually exclusive; **not asked – self disclosed; NR: Not Reported

### Delphi process

**Figure 2.**
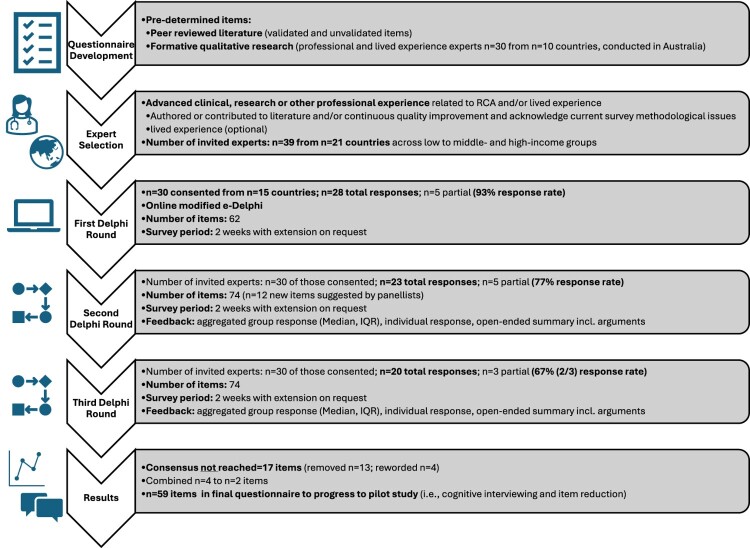
Delphi process

Of the 30 experts consented, the number of experts participating in the first Delphi round was 28, in the second round 23, and in the final third round 20. A couple of experts did not attempt their first-round surveys but did complete surveys in subsequent rounds, and as expected, some experts dropped off after each round. This corresponds to a response rate of 93%, 77%, and 67% (or 2/3) respectively. Figure 2 depicts the full Delphi process.

### Delphi survey results

**Graph 1.**
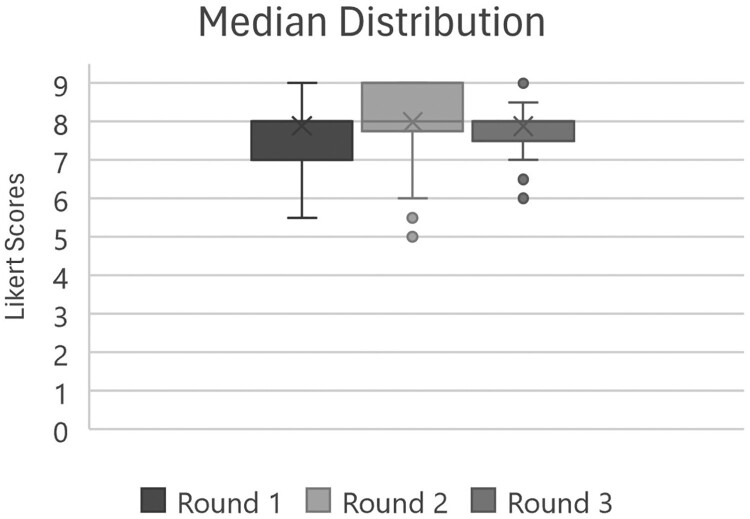
Median distribution of each survey round

**Graph 2.**
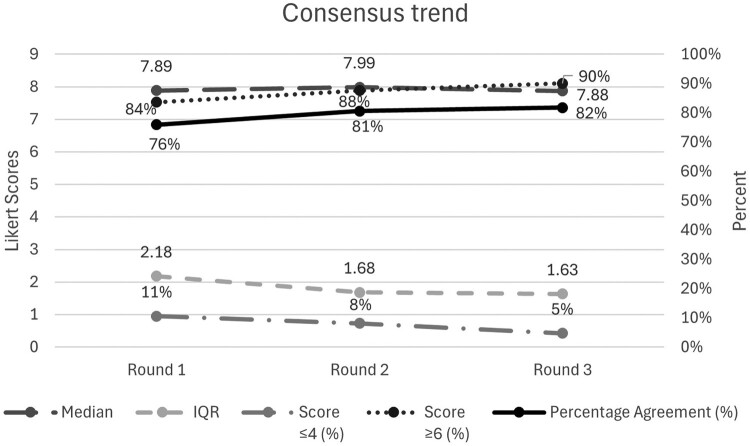
Consensus trend across each survey round 1–3 (averages)

The average percentage agreement across each round increased, as anticipated, from 76% to 81% to 82%. The median distribution of each survey round as well as the consensus trends (averages) are displayed graphically (Graphs 1 and 2). The majority of results were stable across rounds with a statistically significant Wilcoxon value, indicating instability, for *n* = 13 items between Delphi rounds 1–2, reduced to 3 items between rounds 2–3. Only two participants had differences in their median response scores between rounds (one round each) that deviated from 0, indicating overall high stability. Of the items that did not achieve consensus (*n* = 17), some that achieved consensus on relevance but not comprehensibility were reworded (*n* = 4), and the rest removed (*n* = 13). Per panellist suggestions, four items were combined into two items resulting in a final 59 items to progress to pilot testing and psychometric evaluation with the aim of final item reduction. The domains and final item wording that remain prior to factor analysis are summarised below in Table 3. Consensus could not be reached on whether to ask separately about the intent or motivation behind controlling behaviours or how best to structure response options. Therefore, these aspects of the scale's design will await pilot testing. Full Delphi results for each measurement item per round are available in the supplemental files (Supplemental file 1), along with the final item outcomes and a summary of free-text responses (Supplemental file 2).

**Table 3.** Domains and items prior to factor analysis

Domain	Final items (pre-pilot testing)
Pregnancy Coercion & Abuse	I felt coerced or forced by another person to become pregnant very soon after a previous pregnancy or pregnancy loss *(e.g. < 12 months; when I was not ready and/or medically advised not to; after delivering a girl child).*
	I felt coerced or forced by another person to do things they thought would make me get pregnant, when I did not want to have a baby *(e.g. stay in different positions during or after sex; eat or drink specific things; take part in ceremonies or rituals)*.
	Another person coerced or forced me to have more children than I wanted *(e.g. wanted to stop at 2, but had to have 3; wanted 0, but had to have 1, etc.).*
	I was threatened that if I did not get pregnant when someone else wanted, they would do bad things^1^ to me or my loved ones.
	I felt coerced or forced to take medication so I could become pregnant when I did not want to *(e.g. hormones, injections, IVF/fertility treatment; medicine to start puberty).*
	I was treated badly by another person, because it was difficult for me to become pregnant and/or give birth to a live baby in the past *(e.g. abandoned, made to feel worthless)*.
	Another person tried to coerce or force me into not receiving gender-affirming care, so that I could get pregnant (e.g. not use hormones or other medicines like puberty blockers, testosterone, etc.).*
	I was treated badly by another person for not wanting to be pregnant *(e.g. abandoned, made to feel worthless)*.*
	I felt coerced or forced by another person to become pregnant immediately after marriage when I did not want to or did not feel ready.*
Manipulation/ Blackmail	I was threatened a sex/romantic partner would leave me or have a baby with someone else if I did not get pregnant *(e.g. I will be divorced and/or they will take another wife/partner, etc.).*
	I was threatened that another person would harm themselves (e.g. suicide) if I did not make the pregnancy choice they wanted *(e.g. continuing or ending a pregnancy).*
	I was told I had to have a baby to prove that I was a “good” wife/partner, daughter/child, or in-law.*
Sexual Violence Overlap	I felt pressured or forced by another person to have sex *(e.g. more sex than I wanted). If yes*, I think this was because the person was trying to get me pregnant.
	Someone I had sex with ejaculated (i.e. ‘came’) inside me before pulling out their penis even though they told me, or I thought, they would.*If yes*, I think this was because they were trying to get me pregnant.
	A person I had sex with pressured or forced me to have sex without a condom *(e.g. refused to use a condom; would not put a new condom on after one broke or came off; tricked me to think they were wearing a condom when they were not).*
	Following unwanted or forced sex, I felt pressured or forced by another person to take emergency contraception or to have an abortion.*
Contraception Coercion & Abuse Use	When I wanted to stop using contraception, I was prevented from stopping it or getting care to *(e.g. money/transport withheld, lied to/phone monitored to disrupt appointment when trying to have a contraceptive device like an IUD removed or get advice about stopping the ‘pill’).*
	I have been tricked into using contraception without my knowledge or permission *(e.g. sneaking pills in my food or drink; receiving contraception without knowing what it was; IUD inserted after childbirth).*
	I felt coerced or forced to go to the health clinic, pharmacy, etc. to use contraception that I did not want to use *(e.g. appointment booked on my behalf; taken to get the ‘pill’ or have a contraceptive device like an IUD inserted)*.*
Contraception Coercion & Abuse Non-Use	I was threatened someone would do bad things^1^ to me or my loved ones if I used contraception*. If yes,* did you keep it a secret that you used or wanted to use contraception?
	I wanted to stop or change my contraception method, but another person did not let me. *If yes,* how likely do you think it was that they wanted to control whether you became pregnant?
	I felt coerced or forced by another person to have a contraceptive device removed *(e.g. to visit health clinic to remove IUD or implant (“bar/rod”); they forcibly removed it from my body).*
	I was prevented from going to a health clinic, pharmacy or other store to get contraception *(e.g. money/transport withheld, lied to/phone monitored to disrupt appointment).*
	I was given false information to keep me from using contraception *(e.g. told we did not have enough money, the clinic was out of stock, or contraception has dangerous side effects/causes infertility).*
	Another person refused to let me use contraception, although they knew I did not want to become pregnant.
	My contraception was interfered with or destroyed by another person *(e.g. pills or injections hidden, thrown away, flushed down the toilet, or replaced with fake contraception to trick me; poked holes in condom).*
	I was denied contraception due to another person's individual or religious beliefs *(e.g. I need my partner's permission; I should start a family; I’m too young; it is against God's will; means I have more than one sex partner).*
	Another person refused to let me use the type of contraception I wanted to use (e.g. I could use condoms, but not the ‘pill’ or IUD).*
Coercive/ Micro-control	My confidence in my reproductive health decisions was undermined by another person who constantly questioned my choices *(e.g. to use contraception, become pregnant, keep or end a pregnancy).*
	Another person came with me to a medical appointment to stop me from speaking freely about my pregnancy and/or contraceptive choices *(e.g. to watch over me; not letting me have a professional interpreter; for fear I would terminate a pregnancy)*.
Pregnancy Outcome Interference – Bidirectional	I was harassed or stalked by another person to find out whether I was going to keep or end a pregnancy *(e.g. constant calling, texting with angry tone).*
	I was told I would cause another person's distress or poor health if I did not fulfil their wish about a pregnancy (e.g. continuing or ending the pregnancy).*
Abortion Coercion & Abuse Coerced/ Forced Pregnancy	My pregnancy news was shared with another person before I was ready, and this limited my pregnancy options *(e.g. told to my in laws/partner's family so I could not get an abortion without them finding out).*
	I changed my decision from ending a pregnancy to keeping it because of constant, unwavering pressure from another person.
	I was delayed or stopped by another person from ending a pregnancy (i.e. abortion) *(e.g. money/transport withheld, lied to/phone monitored to disrupt health care appointment).*
	I ended a pregnancy (i.e. abortion) and kept it a secret, because another person threatened to do bad things^1^ to me or my loved ones*.*
	To convince me not to end a pregnancy (i.e. abortion), I was coerced or forced to see a religious or cultural leader *(e.g. priest, elder).*
	To stop me from ending a pregnancy, I was told other people would be informed if I had an abortion *(e.g. my family, employer, police/ legal authorities, etc.).*
	I continued a pregnancy, because of another person's threats to do bad things^1^ to me or my loved ones if I had an abortion.
	I was not allowed to end a pregnancy (i.e. abortion) due to another person's individual or religious beliefs *(e.g. I need my partner's permission; I should start a family; I’m too young; it is against God's will)*.*
Abortion Coercion & Abuse Coerced/Forced Abortion	I changed my decision from keeping a pregnancy to ending it (i.e. abortion) because of constant, unwavering pressure from another person.
	I felt coerced or forced into ending a pregnancy (i.e. abortion) by another person *(e.g. when I did not want one, was not ready, or not sure).*
	I was refused financial and caregiving support to make me feel like I must have an abortion *(e.g. having to care for the baby by myself once born).*
	I was threatened another person would do bad things^1^ to me or my loved ones if I kept the pregnancy.
	I or my loved ones were harmed by another person, because I did not have an abortion when they wanted me to *(e.g. physically harmed; relationship ended; disowned from family or religion; refused money/housing; reputation ruined; reveal private or damaging information about me to others, visa canceled)*.*
	I was given medicines to cause a miscarriage without my knowledge or permission (e.g. sneaking it in my food or drink, receiving it without knowing what it was).*
	I was coerced or forced to see a religious or cultural leader when pregnant to convince me to end a pregnancy (i.e. abortion).*
Selective Reproduction	I felt coerced or forced by another person to end a pregnancy (i.e. abortion), because the baby might have a health problem or disability *(e.g. genetic disorder; life-limiting illness).*
	I was made to feel guilty and worthless by another person if I did not have a baby of a certain sex *(e.g. male or female).*
	I felt coerced or forced by another person to continue to get pregnant until I gave birth to a child of a certain sex *(e.g. male or female).*
	I felt coerced or forced by another person to end a pregnancy (i.e. abortion), because the f(o)etus was not of a certain sex *(e.g. male or female).*
Fertility Preservation – Permanent Contraception – Other	I was delayed or prevented from accessing life-saving health care by another person, due to the impacts it might have on my fertility *(e.g. cancer treatment, gender affirming care).*
	I was stopped from having a permanent birth control procedure *(e.g. tubes tied, hysterectomy)* when I wanted one, because another person thought I should have a baby *(e.g. told I am too young or will regret it; money/transport withheld; lied to/phone monitored to disrupt appointment).*
	I felt coerced or forced to have a permanent birth control procedure *(e.g. tubes tied, hysterectomy)* when I did not want one.
	Another person tried to guilt me into not getting pregnant by constantly saying I would never be a good parent *(e.g. because of my mental health, disability, gender and/or sexuality).*
	I was threatened with having my children taken away from me if I had another baby.
	I was physically harmed by another person who wanted to end my pregnancy (e.g. beaten, kicked/ punched in the stomach, thrown, unwanted rough sex, etc.).

^1^ *bad things* refers to e.g. physically harm; end the relationship; disown from family or religion; refuse money/housing; ruin reputation; reveal private or damaging information about me to others; cancel my visa; *panel suggested item

Notes: Add/substitute “menstrual regulation” for abortion where contextually relevant. For sex selection items, can have a follow up item for the person to choose the preferred sex.

#### Categories of actors

Panellist experts were asked to consider the multitude of RCA actors in line with expanding conceptualisations of reproductive autonomy, including contraceptive agency, calling for broadened acknowledgment and measurement of interference sources beyond intimate partners.^55^ Free text responses indicated consensus that interpersonal abuse inflicted within relationships where the actor has influence over the individual's or couple's reproductive health decisions was distinct from general cultural and societal expectations and norms, as well as institutional and societal barriers (e.g. restrictive SRHR policies/laws; limited SRH care supplies/infrastructure). Free-text responses indicated that these threats to reproductive autonomy that can normalise or foster abuse should be measured separately, either through their own items underneath subdomains or preferably a separate instrument.

Categories that reached consensus for inclusion within our measure of interpersonal RCA were:

- Current sexual/romantic partner(s) (such as boyfriend/girlfriend, husband/wife, casual hook-up, etc.)
- Past sexual/romantic partner(s) (such as boyfriend/girlfriend, husband/wife, casual hook-up, etc.)
- Parent(s)/guardian(s), please list ____________
- My own family (excluding parents) (such as grandmother, brother, etc.), please list _____________________
- In-laws (my partner's family, such as mother-in-law, sister-in-law, etc.), please list _____________________

- Health or social service professional (such as doctor, nurse/midwife, carer, support worker/case manager, etc.)
- Other person(s) with authority in your life, please list _______________ (such as religious leader, elder/respected community member, teacher, coach etc.)

#### Results regarding wording and comprehensibility

Panellists were presented with different question styles to elicit their opinions based on findings from our qualitative research (e.g. indirect questioning to accommodate high context cultures where “saving face” is important and information is communicated through implicit meanings and non-verbal cues).^56^ Question styles included indirect, passive voice (e.g. I was lied to), subjective items (i.e. feeling/impact centred) (e.g. I felt lied to), and direct, behaviourally specific items (e.g. Someone lied to me). Differing opinions were expressed. Several experts preferred direct, behaviourally specific items, noting this question style is recommended for high quality GBV measurement and can help simplify item wording, finding indirect questions were prone to convoluted and confusing wording^39^

## Discussion

In consultation with global experts, we developed a new RCA measure. The central finding from our study is that there is global consensus on what meets criteria for RCA that expands on previous scales and which key behaviours can be measured across multi-country contexts. By expanding on prior work, the scale reported on here may address current challenges in RCA measurement. The scale we developed benefits greatly from and builds on the seminal work of Miller et al. and the substantive research conducted since 2010 using the Reproductive Coercion Scale across global contexts.^2,12^ In their recent research, developers of the Reproductive Coercion Scale found that when the full measure's pregnancy coercion sub-scale was adapted globally, the most common RCA behaviours were not the same across multi-country study sites.^12^ The authors suggested that the single item used in the Demographic Health Survey (DHS) to broadly measure RCA, derived from the Reproductive Coercion Scale, may have low sensitivity and underestimate prevalence in large-scale surveys. Further, they raised that limited condom use in some countries, or primary use for prevention of sexually transmitted infections (i.e. not pregnancy prevention), limits the ability to implement the Reproductive Coercion Scale in full, including its short form.^57,58^ By comparison, our scale leverages the knowledge of a wide range of diverse country-specific as well as generalised expertise and considers traditional family planning methods (e.g. withdrawal), in addition to condom use.

Whilst additional research is required before the scale developed here is ready for wider use (e.g. cognitive testing, item reduction through psychometric evaluation, translation), we believe the findings are generalisable to global contexts given the diversity of expertise contributing to the scale's development. The items generated come from global literature, including all conceptual research to date about RCA and its related concept, reproductive autonomy. Candidate items were further underpinned by diverse lived expertise (i.e. victim-survivor centred) identified during our formative phase from partnered and unpartnered women and adolescents from multiple countries, uncommon in SRH measurement development.^59^ Lived experience voices were additionally bolstered by practitioner perspectives. Item content validity was then strengthened by global knowledge attained through decades of research and clinical practice contributed by panellist experts during the Delphi study.

Towards advancing SRHR, our research also set out to address some of the conceptual and structural factors challenging how we measure sexual empowerment and agency in family planning, particularly how to balance between context speciﬁcity and multi-country comparability.^19,59^ On behalf of the family planning measurement group, Bhan et al. (2022) argue striking the balance *“requires communication and partnerships between implementers and a wider stakeholder group for greater harmonisation of measures across contexts and for use within family planning programmes”.*^19^ Our development phases incorporated broad stakeholder input. The Delphi panel purposively included researchers and participants with applied experience in SRH and GBV programming and clinical practice. Participant demographics spanned broad geographic regions, as well as professional and personal backgrounds. By measuring broader types of actors, our measure focuses on interpersonal RCA whilst considering the broader interactional context of reproductive health decision-making, such as family obligations and health systems (e.g. conscientious objection). Hinson et al. argue for inclusion of “what matters” beyond “who decides” in reproductive health decisions, for example, whether women share their contraceptive and childbearing preferences, feel valued, and are satisfied with their level of participation in those decisions.^19,60^ While Hinson's measure, developed and validated in Nepal, informed our measure, among others, our items about communication during the Delphi study dropped out as they did not reach consensus. Our Delphi results aligned with our earlier development phases that found that in a context of abuse, responses to these types of items may be *“distorted”,* ultimately leading us to remove such items from our measure.^60^ By contrast, some revisions of the Reproductive Coercion Scale intended for clinical use in the US have added items on communication, finding its inclusion beneficial.^61^ While further refinement is needed, we hope our new measure will contribute to better understanding of RCA across global contexts, most importantly how it can be prevented and reduced, current research equity issues. Our research offers an RCA outcome measure that upholds SRHR by prioritising reproductive choice and agency as outcomes in themselves, described by Bhan et al. (2022) as *“an urgent need”* that *“may hold the key to strengthening family planning programmes, address unmet [SRH] needs and deliver effective family planning services to communities”*.^19^

### Strengths and limitations

While 13 (43%) of our participants worked in low- to middle-income countries, a vast majority (85%) of our total sample resided in high-income countries. This included half of those born in low-to middle-income countries, 10 (almost 40%) of the total sample, of whom all remained connected to and worked in their countries of origin. While there are unique strengths to global citizen perspectives and cross-cultural skillsets, that privilege is not afforded to most of the world's populations. It is a limitation that we were unable to include participants resident in select countries within South or East Asia, the Arab peninsula, or Latin America, given we intended for our measure to capture nuanced cultural considerations, some of which may be temporal as social norms can shift over time. In some geographical regions, related studies recruiting at a similar time to ours may have affected recruitment success by contributing to research fatigue among a limited pool of content experts. We were also not successful in recruiting more cisgender men or transgender and gender-diverse panellists, despite recruitment efforts. While some items within our measure may apply to people assigned male at birth, the scale was developed specifically for people with current or past capacity to become pregnant, including cisgender women, transgender and gender-diverse individuals, where the risk of GBV and RCA is highest. Our measure is inclusive of people who experience infertility or have undergone sterilisation procedures. Our anonymisation approach had several strengths, notably ensuring confidentiality, which allowed participants to disclose personal experiences of abuse. While anonymity is a vital element of traditional Delphi methodology, there are also potential limitations.^44^ For example, real-time discussions could have resolved some rewording issues or other debates such as how best to ask about intent. Responses in the free-text comments were analysed by one author, which may have affected the reliability of the results.

It is worth noting that the Delphi surveys were run against the backdrop of the 2024 USA presidential election campaign, as well as numerous other countries’ elections where reproductive rights were on the ballot.^62^ With the USA as a major global donor to family planning programmes with corresponding influence, reproductive autonomy was routinely debated across global news outlets, social media platforms, other public forums, and among the SRHR sector. The simultaneous political discussion could have influenced experts’ responses by, for example, raising awareness about particular issues or altering beliefs and attitudes.^63^

### Future research

To deal with discrepancies in expert preferences regarding item structure (e.g. how to ask about intent), we seek to empirically test and compare in parallel the performance of different versions of the scale items during subsequent validation phases. Future pilot testing (e.g. cognitive interviewing) with low literacy populations and in specific geographical locations is essential to ensure the measure's overall reliability and validity (e.g. in a sample of nations across income groups and diverse cultural contexts). Readability challenges remain for some of the measure's items despite wording changes across survey rounds to increase their comprehensibility. While their meaning was understood by panel participants, a higher literacy level is required. For example, the move to use “coercion” as a more precise term to “pressure”, following iterative Delphi rounds, will require sufficient cognitive testing and wordsmithing with diverse populations as “coercion” did not test well in our qualitative phase. Future refinement will focus on psychometric evaluation of the full research scale, including item reduction as well as validating a short-form of clinically meaningful questions to identify and respond to RCA in health care settings. We aim, through future validation studies across contexts, to identify a minimum core set of items and behaviours for multi-country comparison with supplemental items, or sub-domains, for researchers and policy makers seeking more granularity. The minimum core set will be selected from items which perform the best during cognitive and psychometric testing across the majority of future sample sites.

## Conclusion

Together with globally recognised experts, we developed a robust set of new items to measure RCA that fills a research gap in sexual and reproductive health measurement. With future psychometric evaluation, the scale will enable more sensitive, specific, and consistent measurement of interpersonal RCA to estimate prevalence, identify risk factors, and evaluate interventions within multi-country comparative studies. A more comprehensive understanding of RCA is critical to develop targeted, evidence informed practice and policy solutions locally and globally.

## Supplementary Material

Supplemental Material File 2: Final items

Supplemental Material File 1: Delphi Results

Delphistar

## Data Availability

We did not receive consent to share individual participant data. Aggregated data is available in the manuscript and supplemental files. Other study materials are available from the authors upon reasonable request.
