# Supplementary material for: Development of a comprehensive measure of reproductive coercion and abuse for global use: a Delphi study
Source: Sex Reprod Health Matters. 2026 Apr 8;33(1):2652218. doi: 10.1080/26410397.2026.2652218 (PMC13206697; doi:10.1080/26410397.2026.2652218)
Supplement: Supplemental Material File 2: Final items [file ZRHM_A_2652218_SM0626.pdf]

Domain	#	**Final item wording** (pre-pilot testing)	**Feedback summary**	**Consensus**	**Outcome**
PREGNANCY COERCION & ABUSE	1	Another person watched me closely in a way that felt uncomfortable and intrusive to see whether I was fertile or pregnant *(e.g., tracking my period/menstruation/ovulation cycle, how often I had sex, body changes, etc.).*	may be an invasion of privacy more than abuse but may suggest pattern of behaviours or early signs of abuse; culturally relative, global scale should be targeted to only capture actual RC/RV	No (Comp only)	Remove
	2	I felt coerced or forced by another person to become pregnant very soon after a previous pregnancy or pregnancy loss *(e.g., < 12 months; when I was not ready and/or medically advised not to; after delivering a girl child).*	dropping 'pressure' may miss women who don't feel they were 'forced' per se; 'coerced' for some contexts not easily understood; some prefer 'having a baby' over 'previous pregnancy;' readability may be high	Yes	Interrogate 'coerce' and 'previous pregnancy' in cognitive interview phase
	3	I felt coerced or forced by another person to do things they thought would make me get pregnant, when I did not want to have a baby *(e.g., stay in different positions during or after sex; eat or drink specific things; take part in ceremonies or rituals).*	disagreement whether 'when I did not want to have a baby' is needed (may be unsure; force implies); can customise 'things' based on context; some prefer 'acts-based- items over subjective impact/feeling, others disagree	Yes	Interrogate different wording options; consider when not trying to get pregnant
	4	Another person coerced or forced me to have more children than I wanted *(e.g., wanted to stop at 2, but had to have 3; wanted 0, but had to have 1, etc.).*	requests to separate no children from fewer; insinuates 'giving children back'	Yes	Not stable across rounds 2 & 3; retain per qual. phase results
	5	I was threatened that if I did not get pregnant when someone else wanted, they would do bad things to me or my loved ones *(e.g. physically harm; end the relationship; disown from family or religion; refuse money/housing; ruin reputation; reveal private or damaging information about me to others; cancel my visa).*	Differ in preference of afraid vs threatened; consider separating out harming loved ones; intertwines structural and interpersonal consequences	Yes	Cognitively test incl. loved ones; test 2 styles of question orientation
	6	I felt coerced or forced to take medication so I could become pregnant when I did not want to *(e.g., hormones, injections, IVF/fertility treatment; medicine to start puberty).*	May be rare, or context specific; some prefer I was vs. I felt	Yes	Test 2 styles of questions active vs. subjective feeling
	7	I was treated badly by another person *(e.g., abandoned, made to feel worthless)*, because it was difficult for me to become pregnant and/or give birth to a live baby in the past.		Yes	
PREGNANCY COERCION & ABUSE – MANIPULATION/ BLACKMAIL	8	I was threatened if I did not get pregnant or have a baby *(e.g. to be disowned/kicked out of my family; shamed in my community; denied food; not allowed to visit my family, accused of being a witch or having sinned in my faith; etc.).*	Revert to round 2 and add other "I was threatened if I do not get pregnant or have a baby, I would be disowned, kicked out of my family, or other."	No (Comp only)	Remove
	9	Another person tried to blackmail me into having children by making me feel like I was worthless for not fulfilling my childbearing role *(e.g., told me I am responsible for providing a baby to continue the family bloodline; provide grandchildren; care for family, business or inheritance).*	confusing, person may not know intent; change to pressure; too specific/complex; does not meet definition of blackmail; overlaps with cultural/social pressure - emotionally difficult, but not necessarily abuse	No	Remove

	10	I was lied to by a sex/romantic partner who said that they were not able to have children *(e.g., led to believe they were infertile or had a sterilisation procedure like a vasectomy).*	may be too specific; wordy; include 'trick', e.g. "A sexual/romantic partner pretended to be infertile to trick me into getting pregnant"	No	Remove
	11	I was threatened a sex/romantic partner would leave me or have a baby with someone else if I did not get pregnant *(e.g., I will be divorced and/or they will take another wife/partner, etc.).*		Yes	
	12	I was blackmailed by suggesting I did not love my sexual/romantic partner if I refused to have a baby with them.	threaten not accurate word; need to include similar example with abortion; blackmail challenging word	No (Comp only)	Remove – blackmail embedded in other item examples
	13	I felt worthless when another person told me my life had no value if I did not have a baby *(e.g., first child or another baby).*	Coercion regardless of feeling (may underrepresent); double barreled; repronormativity/social norm vs abuse	No	Remove
	14	I was promised financial and caregiving support if I kept a pregnancy when I did not want, or was not ready, to have a baby *(e.g., "I will pay you $x to continue the pregnancy"; "I will help you with everything you need, no matter what").*	support is withdrawn may be more important; too specific; blackmail example	No	Remove
	15	I was threatened that another person would harm themselves (e.g., suicide) if I did not make the pregnancy choice they wanted *(e.g. continuing or ending a pregnancy).*		Yes	
**PREGNANCY COERCION & ABUSE - SEXUAL VIOLENCE OVERLAP**	16	Part A) I felt pressured or forced by another person to have sex (e.g., more sex than I wanted).<br>Part B) I think this was because the person was trying to get me pregnant.		Yes	
	17	After being pressured or forced to have sex, I had to marry and keep the baby to cover up that they were conceived due to unwanted, premarital sex.	Sexual assault/ forced marriage not RC/ conflated; not clear to all how marriage/having baby covers up 'baby born of rape'	No	Remove
	18	Part A) Someone I had sex with ejaculated (i.e. 'came') inside me before pulling out their penis even though they told me, or I thought, they would.<br>Part B) I think this was because they were trying to get me pregnant.		Yes	
	19	Part A) A person I had sex with pressured or forced me to have sex without a condom *(e.g., refuse to use a condom).*<br>Part B) I think they were trying to get me pregnant.	Concerns how part A and part B can be analysed using factor analysis	Yes	Combine 19&20
	20	Part A) A person I had sex with pressured or forced me to keep having sex after the condom came off or broke *(e.g. taken off or broken intentionally or unintentionally).*<br>Part B) I think they were trying to get me pregnant.	Combine with 19; in some countries condom questions may not be required if not widely available (e.g., catholic nations); not clear includes poking holes (incl. item 31)	Yes	
			Combined item (19/20): A person I had sex with pressured or forced me to have sex without a condom *(e.g., refuse to use a condom; would not put a new condom on after one broke or came off; tricked me to think they were wearing a condom when they were not).*		

**CONTRACEPTION COERCION & ABUSE – USE**	21	When I wanted to stop using contraception, I was prevented from stopping it or getting care to *(e.g., money/transport withheld, lied to/phone monitored to disrupt appointment when trying to have a contraceptive device like an IUD removed or get advice about stopping the 'pill').*		Yes	
	22	I have been tricked into using contraception without my knowledge or permission *(e.g., sneaking pills in my food or drink; receiving contraception without knowing what it was; IUD inserted after childbirth).*		Yes	
**CONTRACEPTION COERCION & ABUSE – NON – USE**	23	I was threatened someone would do bad things to me or my loved ones if I used contraception *(e.g. physically harm; end the relationship; disown from family or religion; refuse money/housing; ruin reputation; reveal private or damaging information about me to others; cancel my visa).*		Yes	
	24	I kept it a secret that I used or wanted to use contraception, because another person threatened to do bad things to me or my loved ones *(e.g. physically harm; end the relationship; disown from family or religion; refuse money/housing; ruin reputation; reveal private or damaging information about me to others; cancel my visa).*	Same as above afraid vs. threatened; could have as a follow-up to 23 to measure harm reduction strategies	Yes	Consider as follow-up to 23
	25	I wanted to stop or change my contraception method, but another person did not let me.	Motive unclear	Yes	Consider follow-up item for intent
	26	I felt coerced or forced by another person to have a contraceptive device removed (e.g., IUD or implant ("bar/rod")).	Could combine with 30; remove health clinic location; direct act vs. feeling	Yes	Combine with 30
	27	I was prevented from going to a health clinic, pharmacy or other store to get contraception *(e.g., money/transport withheld, lied to/phone monitored to disrupt appointment).*		Yes	
	28	I feel like I was lied to in order to prevent me from using contraception *(e.g., being told we do not have enough money, the clinic was out of stock, or contraception has dangerous side effects/causes infertility).*	Direct lied to vs subjective feel; suggest wording "I was given misinformation to keep me from using contraception"	Yes	Reword; Pilot lied to vs feel lied to
	29	Another person refused to let me use contraception, although they knew I did not want to become pregnant.		Yes	
	30	Another person forcibly removed a contraceptive device from my body *(e.g., an implant ("bar/rod"), IUD; or ring/patch).*			Combine with 26
			Combined item (26/30): "I felt coerced or forced by another person to have a contraceptive device removed *(e.g., to visit health clinic to remove IUD or implant ("bar/rod"); they forcibly removed it from my body).*		
	31	My contraception was interfered with or destroyed by another person *(e.g., pills or injections hidden, thrown away, flushed down the toilet, or replaced with fake contraception to trick me; poked holes in condom).*	Interfered may be challenging	Yes	Cognitively test interfere
	32	I was denied contraception due to another person's individual or religious beliefs *(e.g., I need my partner's permission; I should start a family; I'm too young; it is against God's will; means I have more than one sex partner).*		Yes	
**COERCIVE/ MICRO CONTROL**	33	My confidence in my decisions about using contraception or becoming pregnant was undermined by another person who constantly questioned my choices.	Complex; not all agree RCA; could make clearer RCA by saying "questioned my decisions"	No	Reword; Cognitively test
	34	My confidence in my decision to keep or end a pregnancy (i.e., abortion*) was undermined by another person who constantly questioned my choices.	complex; overlap with 33; not all agree RCA; measures unique aspect of RCA if combined with other items	Yes	Cognitively test

	35	Another person came with me to a medical appointment to stop me from speaking freely about my pregnancy and/or contraceptive choices *(e.g., to watch over me; not letting me have a professional interpreter; for fear I would terminate a pregnancy).*		Yes	
**PREGNANCY OUTCOME INTERFERENCE- BIDIRECTIONAL**	36	I was harassed or stalked by another person to find out whether I was going to keep or end a pregnancy *(e.g. constant calling, texting with angry tone).*		Yes	
**ABORTION COERCION & ABUSE – COERCED or FORCED PREGNANCY**	37	My pregnancy news was shared with another person before I was ready, and this limited my pregnancy options *(e.g., told to my in laws/partner's family so I could not get an abortion without them finding out).*		Yes	
	38	I changed my decision from ending a pregnancy to keeping it because of constant, unwavering pressure from another person.		Yes	
	39	I was delayed or stopped by another person from ending a pregnancy (i.e. abortion) *(e.g., money/transport withheld, lied to/phone monitored to disrupt health care appointment).*		Yes	
	40	I was talked out of ending a pregnancy (i.e., abortion) when I was considering it.	Alternate situations where not coercion	No	Remove
	41	I ended a pregnancy (i.e., abortion) and kept it a secret, because another person threatened to do bad things to me or my loved ones *(e.g. physically harm; end the relationship; disown from family or religion; refuse money/housing; ruin reputation; reveal private or damaging information about me to others; cancel my visa).*	Simpler language	No (Comp only)	Reword
	42	To convince me <u>not</u> to end a pregnancy (i.e., abortion), I was coerced or forced to see a religious or cultural leader *(e.g., priest, elder)*.		Yes	
	43	To stop me from ending a pregnancy, I was told other people would be informed if I had an abortion *(e.g., my family, employer, police/ legal authorities, etc.).*		Yes	
	44	I continued a pregnancy, because of another person's threats to do bad things to me or my loved ones if I had an abortion *(e.g., physically harm; end the relationship; disown from family or religion; refuse money/housing; ruin reputation; reveal private or damaging information about me to others; cancel my visa).*		Yes	
**ABORTION COERCION & ABUSE - COERCED or**	45	I changed my decision from keeping a pregnancy to ending it (i.e., abortion) because of constant, unwavering pressure from another person.		Yes	
	46	I felt coerced or forced into ending a pregnancy (i.e., abortion) by another person *(e.g., when I did not want one, was not ready, or not sure).*		Yes	
	47	Another person tried to coerce, threaten or force me into having an abortion by arguing that their needs were more important than mine *(e.g., because they didn't want to be a parent/father).*	some question whether motivation is relevant for the survey measure	No	Remove

	48	I felt coerced or forced by another person to do things they believed could cause a miscarriage of a pregnancy I wanted *(e.g., stay in different positions during or after sex; eat or drink specific things; take part in ceremonies or rituals).*	questions re. if there is a chance the behaviour does not end pregnancy whether it matters	No	Remove
	49	I was refused financial and caregiving support to make me feel like I must have an abortion *(e.g., having to care for the baby by myself once born).*		Yes	
	50	I was threatened another person would do bad things to me or my loved ones if I kept the pregnancy *(e.g. physically harm; end the relationship; disown from family or religion; refuse money/housing; ruin reputation; reveal private or damaging information about me to others; cancel my visa).*		Yes	
**COMMUNICATION**	51	I feel emotionally and physically safe to have an honest conversation with people who have influence over my pregnancy and/or contraceptive choices.	not all agree RCA; some think most relevant to intimate partner	No	Remove
**SELECTIVE REPRODUCTION****	52	I felt coerced or forced by another person to end a pregnancy (i.e. abortion), because the baby might have a health problem or disability *(e.g., genetic disorder; life-limiting illness).*	Consistent use of baby or f(o)etus	Yes	Cognitively test language baby vs. f(o)etus
	53	I was made to feel guilty and worthless by another person if I did not have a baby of a certain sex *(e.g., male or female).*		Yes	
	54	I felt coerced or forced by another person to continue to get pregnant until I gave birth to a child of a certain sex (e.g., male or female).		Yes	
	55	I felt coerced or forced by another person to end a pregnancy (i.e. abortion), because the f(o)etus was not of a certain sex (e.g., male or female).		Yes	
	56	I felt coerced or forced by another person to do things they believed would increase the chance of having a child of a certain sex *(e.g., male or female) (e.g., stay in different positions during or after sex; eat or drink specific things; take part in ceremonies or rituals).*	some question if behaviour not effective whether it counts; wordy; 54/56 preferred; not all agree RCA	No	Remove
**FERTILITY PRESERVATION - PERMANENT CONTRACEPTION**	57	I was delayed or prevented from accessing life-saving health care by another person, due to the impacts it might have on my fertility *(e.g., cancer treatment, gender affirming care).*	not all agree RCA although a rights violation; others strongly agree; could include forcing to receive additional care to track fertility (captured above)	No (Comp only)	Reword
	58	I was stopped from having a permanent birth control procedure (e.g., tubes tied, hysterectomy) when I wanted one, because another person thought I should have a baby *(e.g., told I am too young or will regret it; money/transport withheld; lied to/phone monitored to disrupt appointment).*		Yes	
	59	I felt coerced or forced to have a permanent birth control procedure (e.g., tubes tied, hysterectomy) when I did not want one.		Yes	
	60	Another person tried to guilt me into not getting pregnant by constantly saying I would never be a good parent *(e.g., because of my mental health, disability, gender and/or sexuality).*		Yes	
	61	I was threatened with having my children taken away from me if I had another baby.		Yes	

	62	I was physically harmed by another person who wanted to end my pregnancy *(e.g., beaten, kicked/ punched in the stomach, thrown, unwanted rough sex, etc.).*		Yes	
**PANEL SUGGESTED ITEMS**	63	Another person tried to coerce or force me into not receiving gender-affirming care, so that I could get pregnant *(e.g., not use hormones or other medicines like puberty blockers, testosterone, etc.)*	messy wording as written, gender-affirming care may not be understood by all; important to keep a transgender specific item	No (Comp only)	Reword
	64	I was told I would cause another person's distress or poor health if I did not fulfill their wish about a pregnancy *(e.g. continuing or ending the pregnancy).*		Yes	
	65	I was told I had to have a baby to prove that I was a "good" wife/partner, daughter/child, in-law.		Yes	
	66	I was treated badly by another person for not wanting to be pregnant *(e.g., abandoned, made to feel worthless).*		Yes	
	67	I felt coerced or forced by another person to become pregnant immediately after marriage when I did not want to or did not feel ready.		Yes	
	68	Following unwanted or forced sex, I felt pressured or forced by another person to take emergency contraception or to have an abortion.		Yes	
	69	Another person refused to let me use the type of contraception I wanted to use *(e.g., I could use condoms, but not the 'pill' or IUD).*		Yes	
	70	I felt coerced or forced to go to the health clinic, pharmacy, etc. to use contraception that I did not want to use *(e.g., appointment booked on my behalf; taken to get the 'pill' or have a contraceptive device like an IUD inserted).*		Yes	
	71	I was not allowed to end a pregnancy (i.e., abortion) due to another person's individual or religious beliefs *(e.g., I need my partner's permission; I should start a family; I'm too young; it is against God's will).*		Yes	
	72	I or my loved ones were harmed by another person, because I did not have an abortion when they wanted me to *(e.g. physically harmed; relationship ended; disowned from family or religion; refused money/housing; reputation ruined; reveal private or damaging information about me to others, visa canceled).*		Yes	
	73	I was given medicines to cause a miscarriage without my knowledge or permission *(e.g., sneaking it in my food or drink, receiving it without knowing what it was).*		Yes	
	74	I was coerced or forced to see a religious or cultural leader when pregnant to convince me to end a pregnancy (i.e., abortion).		Yes	

Notes: *We will add/substitute "menstrual regulation" for abortion where contextually relevant. **We will have a follow up item for the person to choose the preferred sex.

General: Frame questions from the impact or feeling for the person experiencing the behaviour (preference from qualitative phase) vs active voice orienting the actor first (common preference in Delphi phase); in qualitative phase, "coerce" was a difficult word to comprehend and to interpret, reinforced by the Delphi panel, although most experts preferred coerce compared to "pressure" described as too general (also a qualitative phase finding)
