# Supplementary material for: Development of a comprehensive measure of reproductive coercion and abuse for global use: a Delphi study
Source: Sex Reprod Health Matters. 2026 Apr 8;33(1):2652218. doi: 10.1080/26410397.2026.2652218 (PMC13206697; doi:10.1080/26410397.2026.2652218)
Supplement: Supplemental Material File 1: Delphi Results [file ZRHM_A_2652218_SM0619.pdf]

SUPPLEMENTAL FILE 1

Delphi Results

**Response rate**	**Round 1**	**Round 2**	**Round 3**
Total	n=28	n=23	n=20
partial	n=5	n=5	n=3

Item	Cosmin criteria	Med	IQR	Score ≤4	Score ≥6	PA	Med	IQR	Score ≤4	Score ≥6	PA	W1	p	Med	IQR	Score ≤4	Score ≥6	PA	W2	p	Consensus (Y/N)
Full Measure	Comprehensiveness	8	2	4%	87%	78%	8	1.75	6%	89%	83%	0	0.35	8	2	0%	82%	71%	6	0.85	Y
1	Relevance	8	2.25	11%	86%	75%	8	2.5	13%	75%	75%	15	0.39	8	1.5	11%	89%	84%	25.5	0.06	Y
	Comprehensibility	7	4	32%	64%	54%	8	2	9%	78%	78%	10	0.02*	7	2.5	16%	74%	74%	45	0.29	N
2	Relevance	8	2	4%	96%	82%	8	1.5	9%	83%	74%	5	0.04*	8	1	0%	100%	100%	5	1.00	Y
	Comprehensibility	8	2	0%	100%	82%	9	1	0%	100%	100%	4.5	0.24	9	1	5%	95%	95%	1	1.00	Y
3	Relevance	7	2.5	18%	75%	57%	7	1	0%	100%	96%	10.5	0.09	7	1	5%	90%	85%	2.5	0.46	Y
	Comprehensibility	6	3.25	25%	54%	46%	7	2	9%	87%	74%	4.5	0.04*	8	2	10%	80%	60%	19	0.94	Y
4	Relevance	9	2.25	7%	82%	75%	8	1	4%	83%	78%	5	1.00	8.5	1.25	5%	90%	85%	0	0.03*	Y
	Comprehensibility	6	4.5	36%	57%	43%	7	2	4%	91%	83%	8	0.05	8	2	0%	100%	85%	0	0.01*	Y
5	Relevance	9	1	4%	96%	93%	9	2	17%	78%	65%	4	0.79	8.5	1	0%	95%	90%	1	1.00	Y
	Comprehensibility	8	2.25	11%	86%	75%	9	1	4%	96%	96%	0	0.09	8	1.25	5%	90%	85%	12.5	0.86	Y
6	Relevance	5.5	4	29%	50%	43%	7	1	4%	96%	91%	2	0.03*	7	2	5%	84%	63%	7.5	0.59	Y
	Comprehensibility	8	4	21%	64%	61%	8	4	17%	70%	65%	4	0.20	7	1	5%	84%	79%	15.5	0.77	Y
7	Relevance	7	3	18%	64%	54%	7	3	13%	78%	70%	0	0.01*	7	2	5%	84%	68%	7	0.53	Y
	Comprehensibility	8	2.25	4%	82%	75%	8	3	22%	70%	52%	5	0.29	8	2	5%	89%	84%	3	1.00	Y
8	Relevance	9	2	0%	96%	88%	9	2	4%	91%	87%	4	0.85	8	2	16%	84%	79%	6	0.39	Y
	Comprehensibility	9	2	0%	96%	80%	9	1.75	5%	91%	86%	7.5	0.60	8	4.5	26%	63%	63%	18	0.14	N
9	Relevance	8	3	12%	88%	65%	7.5	1	0%	95%	86%	12.5	0.75	7	2	16%	79%	68%	6	0.40	Y
	Comprehensibility	8	1	8%	88%	80%	7	2	18%	77%	59%	43	0.02*	7	3	21%	58%	58%	12.5	0.86	N
10	Relevance	7	4	15%	69%	69%	8	3.75	27%	64%	59%	9	0.44	8	3	16%	74%	74%	1.5	0.59	N
	Comprehensibility	6	4	31%	62%	46%	6	2.5	18%	77%	73%	20.5	0.86	7	3	21%	74%	58%	2	0.35	N
11	Relevance	9	1	4%	96%	92%	9	3	23%	55%	45%	0	0.37	8	1	0%	100%	100%	1	1.00	Y
	Comprehensibility	9	1	4%	96%	92%	9	1	0%	100%	100%	11.5	0.34	8	1.5	0%	100%	95%	5	0.28	Y
12	Relevance	9	1	4%	88%	85%	9	2	9%	91%	82%	0	0.37	8	2	0%	95%	84%	13	0.67	Y
	Comprehensibility	9	1	4%	92%	92%	8	1	0%	95%	86%	6	0.18	7	2.5	21%	79%	68%	50	0.14	N
13	Relevance	8	2	0%	92%	88%	8	2	14%	82%	77%	8.5	0.75	7	3	0%	79%	58%	10	0.10	N
	Comprehensibility	9	1	0%	96%	96%	8.5	1.75	0%	95%	86%	9	0.78	8	3	16%	84%	53%	19.5	0.07	N
14	Relevance	7	3	15%	69%	58%	7	1	5%	91%	91%	4	0.41	7	3	11%	74%	58%	10.5	1.00	N
	Comprehensibility	7	2	8%	85%	58%	7	3	14%	68%	55%	12.5	0.86	8	2	0%	74%	74%	13	0.67	Y

15	Relevance	7	3.5	15%	73%	65%	7.5	1.75	5%	77%	73%	6	0.39	7	2	5%	95%	95%	0	0.10	Y
	Comprehensibility	7	2.75	19%	69%	54%	7	3	14%	86%	68%	2.5	0.03*	7	2	5%	89%	68%	4	0.20	Y
16	Relevance	9	1	0%	100%	96%	9	2	9%	82%	64%	0	1.00	9	1	5%	95%	89%	0	NA	Y
	Comprehensibility	8	2	0%	92%	88%	8	1	0%	100%	100%	21	0.72	8	2	16%	84%	79%	0	0.17	Y
17	Relevance	7	3	16%	64%	52%	6	2	5%	95%	86%	6	0.85	6	1	16%	79%	47%	8	0.35	Y
	Comprehensibility	6	4	28%	60%	48%	6	3	24%	67%	43%	2	0.17	6	2.5	16%	58%	47%	9	0.44	N
18	Relevance	8	2	12%	84%	76%	8	3	19%	62%	48%	0	0.37	8	1.5	5%	89%	89%	0	NA	Y
	Comprehensibility	7	3	20%	68%	52%	7	1	10%	86%	86%	4	0.20	7	2	16%	79%	58%	1.5	0.59	Y
19	Relevance	9	1	4%	96%	92%	9	2	10%	81%	57%	2	0.77	8	1	0%	100%	95%	0	NA	Y
	Comprehensibility	8	2	8%	88%	80%	8	1	5%	95%	90%	5.5	1.00	8	2	0%	100%	95%	0	0.18	Y
20	Relevance	8	2	12%	88%	80%	9	1	10%	90%	81%	4.5	0.50	8	1.5	5%	95%	89%	4	0.77	Y
	Comprehensibility	7	3	8%	80%	60%	8	1	5%	90%	86%	9	0.44	8	1.5	0%	100%	84%	5	0.28	Y
21	Relevance	8	2	8%	84%	80%	8	2	10%	90%	76%	0	0.10	8	1.5	0%	95%	89%	2	0.35	Y
	Comprehensibility	7	4	28%	64%	56%	7	1	0%	90%	81%	9	0.07	7	1.5	11%	84%	79%	0	0.18	Y
22	Relevance	8	2	12%	88%	84%	8	2	10%	90%	71%	0	0.37	8	1	5%	95%	89%	0	0.37	Y
	Comprehensibility	8	2	4%	92%	88%	8	1	10%	86%	86%	3	1.00	8	2	0%	89%	84%	5.5	1.00	Y
23	Relevance	9	1	0%	100%	100%	9	2	0%	95%	95%	3	1.00	9	1	0%	100%	100%	0	1.00	Y
	Comprehensibility	8	1	4%	92%	80%	8.5	0	5%	95%	95%	6	0.40	8	1	0%	100%	89%	0	0.18	Y
24	Relevance	9	1	0%	100%	96%	9	1	0%	95%	85%	7	0.58	9	2	5%	95%	95%	1	1.00	Y
	Comprehensibility	8	2	8%	88%	84%	9	1.25	5%	90%	90%	6	0.19	8	2	5%	95%	84%	2	0.77	Y
25	Relevance	8	2	8%	88%	80%	8	1	0%	95%	90%	0	0.37	8	2	5%	89%	89%	2.5	1.00	Y
	Comprehensibility	8	3	16%	80%	72%	8	2.25	5%	90%	75%	0	0.10	8	2	0%	95%	84%	0	0.06	Y
26	Relevance	8	2	4%	92%	84%	8	2	15%	85%	80%	0	1.00	8	1	0%	95%	95%	0	0.37	Y
	Comprehensibility	8	2	16%	80%	80%	8	1.25	5%	90%	85%	5	0.14	8	1.5	0%	95%	89%	0	0.09	Y
27	Relevance	9	1	0%	100%	100%	9	1.25	0%	95%	90%	0	1.00	9	1	0%	100%	100%	0	NA	Y
	Comprehensibility	8	1	4%	96%	88%	8	1	0%	100%	100%	2.5	0.42	8	1.5	0%	100%	95%	0	0.37	Y
28	Relevance	8	2	4%	84%	80%	8	1.25	0%	95%	85%	0	0.18	8	1	5%	95%	89%	1.5	1.00	Y
	Comprehensibility	8	2	16%	80%	80%	8	1	5%	95%	90%	6	0.40	8	1.5	16%	84%	79%	1.5	1.00	Y
29	Relevance	8	2	8%	84%	76%	9	1.25	10%	90%	90%	0	0.10	9	1	0%	100%	95%	0	1.00	Y
	Comprehensibility	8	3	12%	80%	72%	9	1	0%	95%	90%	0	0.03*	8	2	5%	95%	95%	0	1.00	Y
30	Relevance	9	1	0%	100%	96%	9	1	0%	95%	95%	0	1.00	9	0.5	0%	100%	100%	0	1.00	Y
	Comprehensibility	8	1	12%	84%	80%	9	0	0%	100%	100%	0	0.03*	9	1	0%	100%	100%	0	0.18	Y
31	Relevance	9	1	0%	100%	100%	9	1	5%	95%	90%	0	0.35	9	0	0%	100%	100%	0	NA	Y
	Comprehensibility	9	1	0%	92%	92%	9	0	0%	100%	100%	4.5	0.59	9	1	0%	100%	100%	1	1.00	Y
32	Relevance	8	2	4%	92%	92%	8	1	0%	100%	100%	5	0.28	8	1.5	0%	95%	95%	0	NA	Y
	Comprehensibility	8	2	8%	88%	80%	8	1	0%	95%	95%	0	0.18	8	2	0%	100%	89%	0	1.00	Y

33	Relevance	7	5	36%	64%	52%	7	2	0%	100%	85%	1	1.00	7	2.5	21%	74%	53%	6	0.85	N
	Comprehensibility	6	4	32%	56%	48%	6	4	37%	63%	53%	19	0.72	7	3	16%	68%	53%	6	0.78	N
34	Relevance	7	5	32%	60%	60%	8	3.5	32%	58%	42%	2	0.77	7	2	16%	74%	63%	6	0.85	Y
	Comprehensibility	6	3	36%	60%	36%	7	4.5	26%	68%	63%	10	0.15	7	1.5	16%	79%	63%	6	0.78	Y
35	Relevance	8	1	0%	92%	92%	9	3	21%	68%	58%	0	0.10	8	1	0%	100%	100%	3	0.35	Y
	Comprehensibility	8	2	16%	80%	76%	8	1	0%	100%	100%	7.5	0.60	8	0.5	0%	95%	95%	4.5	0.25	Y
36	Relevance	8	2	12%	84%	80%	8	2	11%	84%	79%	6	0.40	8	2	0%	94%	89%	3	0.35	Y
	Comprehensibility	7	3	12%	84%	72%	7	1.75	0%	100%	89%	4	0.85	8	1.75	0%	100%	78%	2	0.17	Y
37	Relevance	8	2	12%	88%	76%	8.5	2.5	6%	89%	72%	0	0.10	8	1.75	11%	83%	78%	1	1.00	Y
	Comprehensibility	8	2	0%	96%	76%	8	1	11%	89%	89%	0	0.05	8	1	0%	100%	83%	1	1.00	Y
38	Relevance	9	1	0%	88%	88%	9	2	0%	94%	83%	0	0.37	8	1	0%	100%	89%	3	0.35	Y
	Comprehensibility	9	2	8%	84%	84%	9	1	0%	100%	100%	0	0.10	8	1	0%	94%	94%	7.5	0.42	Y
39	Relevance	9	1	0%	100%	100%	9	0	0%	100%	100%	0	NA	8.5	1	0%	100%	100%	1	1.00	Y
	Comprehensibility	8	1	0%	96%	96%	8.5	1	0%	100%	100%	4	0.85	8	2	0%	100%	89%	2.5	1.00	Y
40	Relevance	8	2	16%	84%	80%	8	1	6%	94%	94%	8	1.00	7	2.75	11%	89%	61%	10	0.59	N
	Comprehensibility	7	3	8%	92%	68%	8	2.75	22%	78%	72%	7.5	0.60	7	1.75	11%	89%	72%	1	1.00	Y
41	Relevance	9	2	4%	88%	84%	9	2.75	11%	89%	72%	1.5	0.27	9	2	6%	89%	89%	0	NA	Y
	Comprehensibility	8	2	8%	84%	80%	8	1	6%	94%	89%	1	0.42	7.5	3	11%	83%	67%	1	1.00	N
42	Relevance	8	2	0%	92%	84%	8.5	2	6%	94%	89%	0	0.37	8	2	0%	100%	89%	1	1.00	Y
	Comprehensibility	8	2	8%	80%	80%	8	1.75	0%	100%	94%	7	1.00	8	2	0%	100%	83%	1	0.42	Y
43	Relevance	8	1	4%	96%	88%	8	2	11%	89%	78%	0	1.00	8	1.75	0%	94%	94%	0	1.00	Y
	Comprehensibility	7	2	24%	76%	60%	7	1	6%	94%	94%	20	0.81	7.5	1	0%	83%	78%	4	0.20	Y
44	Relevance	8	2	8%	88%	80%	9	3.5	22%	72%	56%	0	0.10	8	1	0%	100%	94%	1	1.00	Y
	Comprehensibility	8	3	8%	80%	72%	8	1	6%	94%	94%	0	0.04*	8	1.75	0%	100%	78%	2	0.77	Y
45	Relevance	9	1	12%	84%	84%	9	1.75	0%	100%	78%	0	0.18	8	1	0%	100%	100%	0	NA	Y
	Comprehensibility	8	2	4%	92%	84%	8	1	6%	94%	94%	0	0.18	8	0	0%	100%	94%	1	1.00	Y
46	Relevance	8	2	12%	88%	80%	9	1	0%	100%	94%	0	0.37	9	1	0%	100%	100%	0	0.35	Y
	Comprehensibility	8	2	12%	88%	84%	8.5	1	6%	94%	94%	0	1.00	8	1	0%	100%	89%	0	0.17	Y
47	Relevance	8	3	20%	80%	64%	8	1	6%	94%	89%	2	0.79	8	2.75	0%	89%	67%	4.5	0.59	N
	Comprehensibility	8	1	8%	88%	80%	8.5	3	11%	83%	61%	4	0.77	7	2.75	6%	78%	56%	10	0.10	N
48	Relevance	7	3	20%	68%	60%	7	1.75	6%	89%	78%	2.5	0.11	7	2.75	11%	72%	67%	4	0.85	N
	Comprehensibility	7	3	12%	80%	68%	8	3.5	11%	72%	56%	2	0.17	7.5	2	11%	78%	67%	8.5	0.89	Y
49	Relevance	7	4	12%	72%	64%	8	2.75	6%	89%	72%	3	0.04*	8	1	6%	94%	83%	1.5	1.00	Y
	Comprehensibility	7	4	28%	64%	52%	7	2	6%	89%	83%	4.5	0.50	7.5	2	11%	89%	67%	2	0.17	Y
50	Relevance	8	1	12%	80%	76%	9	2	17%	78%	67%	0	0.10	9	1	0%	100%	100%	3	1.00	Y
	Comprehensibility	8	2	12%	80%	76%	8.5	0.75	11%	89%	89%	0	0.10	8	1.75	0%	100%	89%	3	1.00	Y

51	Relevance	7	4	12%	68%	64%	7	1	0%	94%	94%	5.5	1.00	7	3.75	22%	56%	56%	6	0.85	N
	Comprehensibility	7	3	16%	76%	68%	7	3.5	22%	72%	61%	10	1.00	7	1.75	6%	78%	56%	3	1.00	Y
52	Relevance	9	3	13%	83%	70%	9	2.75	11%	83%	61%	0	0.06	8.5	2	0%	100%	94%	1	1.00	Y
	Comprehensibility	9	1	4%	91%	87%	9	1.75	6%	89%	89%	0	0.17	9	2	0%	100%	89%	0	1.00	Y
53	Relevance	9	1.5	9%	91%	83%	8.5	1	6%	94%	89%	0	0.17	9	1	0%	100%	94%	0	1.00	Y
	Comprehensibility	8	1	4%	91%	91%	8	1.75	6%	89%	89%	0	0.37	8.5	1	0%	94%	94%	0	1.00	Y
54	Relevance	9	1	4%	96%	91%	9	1	6%	94%	94%	1	1.00	9	1	0%	100%	89%	1.5	1.00	Y
	Comprehensibility	8	1.5	9%	87%	87%	9	0.75	6%	94%	94%	0	0.17	9	1	0%	94%	89%	0	0.15	Y
55	Relevance	9	2	4%	87%	87%	9	1	6%	94%	94%	0	0.18	8.5	1	0%	94%	89%	1	1.00	Y
	Comprehensibility	8	1	9%	87%	83%	9	1	6%	94%	94%	0	0.37	8.5	1	0%	94%	89%	0	0.35	Y
56	Relevance	7	4	26%	70%	52%	7	1	6%	94%	94%	2	0.77	7	3.75	6%	72%	67%	2	0.77	N
	Comprehensibility	7	3	13%	78%	65%	8	3.75	22%	72%	56%	5	0.29	8	2.75	0%	89%	72%	1.5	1.00	N
57	Relevance	6	4	30%	57%	48%	5.5	3	6%	78%	67%	8	0.35	6.5	2	17%	61%	50%	2	0.79	Y
	Comprehensibility	6	4	39%	52%	26%	6	3	44%	50%	44%	1.5	0.00*	6	3	33%	56%	44%	21	0.03*	N
58	Relevance	9	1	0%	100%	100%	9	3	17%	61%	44%	2	1.00	8.5	1	0%	100%	94%	0	1.00	Y
	Comprehensibility	8	1.5	4%	91%	87%	8	1	11%	89%	89%	15	0.40	8	1.75	0%	100%	89%	0	0.18	Y
59	Relevance	9	1	9%	91%	87%	9	1	17%	83%	83%	1.5	0.59	9	1	0%	100%	100%	0	NA	Y
	Comprehensibility	8	2	4%	91%	83%	8	1	6%	94%	94%	0	0.07	8	1	0%	100%	89%	0	NA	Y
60	Relevance	8	2	0%	91%	78%	8	1	6%	94%	94%	1.5	0.59	8.5	1	0%	100%	100%	0	1.00	Y
	Comprehensibility	8	2	4%	91%	78%	8	2	6%	89%	89%	3	1.00	8	2	0%	100%	83%	2	0.79	Y
61	Relevance	8	1	4%	91%	87%	8	2	6%	94%	83%	3	1.00	8	1	6%	94%	94%	0	NA	Y
	Comprehensibility	8	1.5	4%	87%	78%	8	1	6%	94%	89%	3.5	0.71	8	1.75	0%	94%	83%	0	0.18	Y
62	Relevance	9	1	0%	100%	100%	9	2.75	11%	83%	72%	0	NA	9	1	0%	100%	100%	0	NA	Y
	Comprehensibility	9	1.5	4%	96%	87%	9	1	6%	94%	94%	0	0.05	8.5	1	0%	100%	94%	0	NA	Y
63	Relevance	N/A	N/A	N/A	N/A	N/A	5.5	1	6%	94%	89%	NA	NA	6	2	17%	50%	50%	2.5	0.46	Y
	Comprehensibility	N/A	N/A	N/A	N/A	N/A	6	3	44%	50%	44%	NA	NA	6	2.75	17%	61%	44%	2.5	0.11	N
64	Relevance	N/A	N/A	N/A	N/A	N/A	7	3.75	33%	56%	33%	NA	NA	7	1.75	0%	89%	72%	6	0.37	Y
	Comprehensibility	N/A	N/A	N/A	N/A	N/A	7	1.75	17%	72%	67%	NA	NA	7	2	0%	83%	67%	1.5	0.59	Y
65	Relevance	N/A	N/A	N/A	N/A	N/A	6	1.75	0%	83%	67%	NA	NA	6.5	1	6%	78%	50%	0	0.17	Y
	Comprehensibility	N/A	N/A	N/A	N/A	N/A	5	2	22%	61%	33%	NA	NA	7.5	2	11%	78%	61%	0	0.01*	Y
66	Relevance	N/A	N/A	N/A	N/A	N/A	7	3	44%	39%	33%	NA	NA	7	1	0%	94%	83%	4	0.85	Y
	Comprehensibility	N/A	N/A	N/A	N/A	N/A	7	1	11%	89%	78%	NA	NA	8	1.75	0%	100%	83%	3	0.23	Y
67	Relevance	N/A	N/A	N/A	N/A	N/A	8	1	0%	100%	94%	NA	NA	8	1	0%	94%	89%	1.5	0.27	Y
	Comprehensibility	N/A	N/A	N/A	N/A	N/A	8	1.75	11%	83%	78%	NA	NA	8	2	0%	94%	83%	1	0.20	Y
68	Relevance	N/A	N/A	N/A	N/A	N/A	8	2	6%	83%	83%	NA	NA	8	1	0%	89%	83%	2	0.79	Y
	Comprehensibility	N/A	N/A	N/A	N/A	N/A	8	0.75	6%	83%	83%	NA	NA	8	2	0%	89%	83%	6	0.85	Y

69	Relevance	N/A	N/A	N/A	N/A	N/A	7.5	1.75	0%	100%	100%	NA	NA	8	2	6%	89%	78%	7.5	1.00	Y
	Comprehensibility	N/A	N/A	N/A	N/A	N/A	8	2.5	0%	94%	72%	NA	NA	8	1	0%	94%	89%	1	1.00	Y
70	Relevance	N/A	N/A	N/A	N/A	N/A	8	1	0%	94%	94%	NA	NA	8	1.5	0%	94%	89%	0	NA	Y
	Comprehensibility	N/A	N/A	N/A	N/A	N/A	8	1	6%	94%	83%	NA	NA	8	1.75	0%	94%	89%	0	0.17	Y
71	Relevance	N/A	N/A	N/A	N/A	N/A	9	0.75	0%	94%	94%	NA	NA	9	1	0%	94%	94%	0	1.00	Y
	Comprehensibility	N/A	N/A	N/A	N/A	N/A	8	1	0%	100%	94%	NA	NA	8	1	0%	94%	89%	0	1.00	Y
72	Relevance	N/A	N/A	N/A	N/A	N/A	8	0.75	0%	100%	94%	NA	NA	8	0.75	0%	100%	94%	0	1.00	Y
	Comprehensibility	N/A	N/A	N/A	N/A	N/A	8	1	0%	89%	89%	NA	NA	8	1	0%	100%	83%	1	1.00	Y
73	Relevance	N/A	N/A	N/A	N/A	N/A	8	0.75	0%	100%	94%	NA	NA	8	0.75	0%	100%	100%	1	1.00	Y
	Comprehensibility	N/A	N/A	N/A	N/A	N/A	8	1	0%	94%	83%	NA	NA	8	1	0%	94%	94%	5	1.00	Y
74	Relevance	N/A	N/A	N/A	N/A	N/A	7.5	1.75	0%	100%	78%	NA	NA	7.5	1.75	11%	78%	72%	3	0.37	Y
	Comprehensibility	N/A	N/A	N/A	N/A	N/A	8	1.75	11%	83%	72%	NA	NA	8	1	0%	94%	89%	1	1.00	Y
Preamble	Abusive actors	8	2	0%	100%	96%	8.5	0.75	0%	94%	94%	21	0.90	8	1	0%	100%	100%	0	NA	Y
Part 1	Relevance	8	1	9%	91%	91%	8	1.75	0%	100%	100%	8	0.35	9	1	6%	94%	94%	0	1.00	Y
	Comprehensibility	7	2.5	17%	74%	57%	8	1	6%	94%	94%	1.5	0.07	8	1	6%	94%	82%	1	1.00	Y
Part 2	Comprehensibility	7	2.5	13%	74%	57%	8	0	6%	94%	83%	0	0.03*	8	1	0%	94%	76%	0	NA	Y
	**AVERAGE**	7.89	2.18	11%	84%	76%	7.99	1.68	8%	88%	81%	-	-	7.88	1.63	5%	90%	82%	-	-	Y

*p < 0.05; Med: Median; PA: Percentage Agreement; W: Wilcoxon test statistic; W N/A= (both round scores are the same)
